# Transcriptome analysis unravels spatiotemporal modulation of phytohormone-pathway expression underlying gibberellin-induced parthenocarpic fruit set in San Pedro-type fig (*Ficus carica* L.)

**DOI:** 10.1186/s12870-018-1318-1

**Published:** 2018-06-01

**Authors:** Lijuan Chai, Peng Chai, Shangwu Chen, Moshe A. Flaishman, Huiqin Ma

**Affiliations:** 1 College of Horticulture, China Agricultural University, Beijing, People’s Republic of China; 2 College of Beijing Laboratory for Food Quality and Safety, College of Food Science and Nutritional Engineering, China Agricultural University, Beijing, People’s Republic of China; 3 Department of Fruit Tree Sciences, Agricultural Research Organization, Volcani Center, Bet-Dagan, Israel

**Keywords:** *Ficus carica*, Gibberellin treatment, Parthenocarpy, Transcriptome analysis, Plant hormone

## Abstract

**Background:**

Gibberellin (GA) treatments can induce parthenocarpy in the main crop of San Pedro-type figs, the native non-parthenocarpic fruit, however, the underlying mechanism is still largely unclear.

**Results:**

In our study, GA_3_ was applied to San Pedro-type fig main crop at anthesis. Sharply increased GA_3_ content was detected in both female flowers and receptacle, along with significantly decreased indole-3-acetic acid (IAA), zeatin and abscisic acid (ABA) levels in female flowers, and increased zeatin peak intensity and earlier ABA peak in receptacles. Transcriptome comparison between control and treatment groups identified more differentially expressed genes (DEGs) in receptacles than in female flowers 2 and 4 days after treatment (DAT); 10 DAT, the number of DEGs became similar in the two tissues. Synchronized changing trends of phytohormone-associated DEGs were observed in female flowers and receptacles with fruit development. Modulation of ethylene and GA signaling and auxin metabolism by exogenous GA_3_ occurred mainly 2 DAT, whereas changes in auxin, cytokinin and ABA signaling occurred mainly 10 DAT. Auxin-, ethylene- and ABA-metabolism and response pathways were largely regulated in the two tissues, mostly 2 and 10 DAT. The major components altering fig phytohormone metabolic and response patterns included downregulated *GA2ox*, *BAS1*, *NCED* and *ACO*, and upregulated *ABA 8′-h* and *AUX/IAA*.

**Conclusions:**

Thus GA-induced parthenocarpy in fig is co-modulated by the female flowers and receptacle, and repression of ABA and ethylene biosynthesis and GA catabolism might be the main forces deflecting abscission and producing fig parthenocarpy.

**Electronic supplementary material:**

The online version of this article (10.1186/s12870-018-1318-1) contains supplementary material, which is available to authorized users.

## Background

Gibberellins (GAs) are involved in almost every aspect of plant growth and development. In plant reproductive processes, GAs regulate floral initiation [1], fruit set and growth [2], and seed maturation and germination [3, 4]. Among the abundant GAs identified to date, only GA_1_, GA_3_, GA_4_ and GA_7_ have been recognized as bioactive [5], and only GA_3_, GA_4_ and/or GA_7_ have been successfully applied for parthenocarpic fruit induction [6].

Parthenocarpy, a highly valuable agronomic characteristic, especially in edible fruit crops, means that fruit set and development can be uncoupled from pollination and fertilization and thus the fruit is seedless, such as in fig, banana, persimmon, pineapple and pear [6]. Parthenocarpic fruit can also be triggered by application of exogenous plant hormones, termed artificial parthenocarpy. Auxin and GAs are the most frequently used hormones in parthenocarpic fruit induction [7, 8]. Other plant hormones, including cytokinin [9], ethylene [10] and abscisic acid (ABA) [11], also regulate or participate in parthenocarpic fruit set and growth.

Parthenocarpy requires the unique and coordinated action of different phytohormones, whereas non-parthenocarpic fruit will senescence and abscise if fertilization is not achieved. Auxin, GA and cytokinin are generally recognized as the major regulators of fruit set. Elevated levels of these hormones in tomato ovaries have been associated with fruit set and early fruit growth [12]. Auxin and GA, and the crosstalk between them, largely modulate pollination-dependent and parthenocarpic fruit set in tomato [13]. Transcriptome comparisons between pollinated and parthenocarpic cucumber fruit have shown crosstalk of auxin, GA and cytokinin at the transcriptional level during parthenocarpic fruit set [14]. Exogenous GA_3_ application significantly modulated the expression patterns of plant hormone metabolism and signaling genes in seedless grape, as determined by transcriptome analysis [15]. Previous studies in several plants have demonstrated that parthenocarpy induced by auxin and cytokinin requires downstream GA biosynthesis. Application of exogenous auxin and cytokinin in grape increased the expression of GA-biosynthesis genes, such as *VvGA3ox1*, and repressed GA-catabolism genes, such as *VvGA2ox3* and *VvGA2ox4*, suggesting that auxin- and cytokinin-induced parthenocarpic fruit set requires enhanced GA biosynthesis [9]. Parthenocarpy induction in tomato by cytokinin depends in part on modulation of GA and indole-3-acetic acid (IAA) metabolism, as reflected by the upregulated expression levels of GA-biosynthesis genes such as *GPS*, *GA20ox* and *GA3ox*, and the IAA-biosynthesis gene *ToFZY*, and downregulation of the GA-inactivation gene *GA2ox* [16]. Auxin-induced parthenocarpic tomato fruit set is partially mediated by GAs, as indicated by the dependence of parthenocarpic fruit formation in the unfertilized *entire* tomato mutant with a defective *SlIAA9* gene on regulation of GA metabolism [17]. *SlIAA9*, a member of the *Aux/IAA* family, is an auxin-signaling repressor that prevents tomato ovary development [18]. Moreover, fruit set in tomato induced by auxin analogue 2,4-D was significantly suppressed by GA-biosynthesis inhibitors [7]. Similar to the situation in *Arabidopsis* and tomato, auxin is the major inducer of fruit set in *Capsicum annuum*, depending in part on enhanced GA biosynthesis [19]. Ethylene is also considered to be a key regulator in coordinating fruit set, and ethylene reduces fruit set by suppressing GA metabolism [10].

*Ficus carica* L. (Moraceae) bears urn-shaped fruit with an enclosed inflorescence structure termed syconium. There are two major sex types in fig trees: caprifig (male fig) and female fig; the latter is further classified into three types, common, Smyrna and San Pedro, based on cropping characteristics [20]. Parthenocarpic or pollinated non-parthenocarpic fig fruit show a typical double-sigmoid growth curve, with two rapid fruit-size-increment phases, i.e., stages I and III, separated by a lag phase (stage II) [21]. Every year, San Pedro fig produces a parthenocarpic first crop called breba, and a non-parthenocarpic main crop which constitutes the main yield [20]. Studies on non-parthenocarpic Smyrna fig cv. Calimyrna revealed that parthenocarpic fruit set can be induced through auxin, GA and cytokinin treatments [22, 23]. Analysis of auxin and GA content in the two crops of San Pedro fig cv. King demonstrated higher auxin content in the parthenocarpic breba than the pollinated non-parthenocarpic main crop, whereas GA was higher in the latter than in the former [24]. Our previous studies revealed that compared with San Pedro parthenocarpic breba, expression of GA- and auxin-biosynthesis genes is repressed in its non-parthenocarpic main crop female flowers, whereas ABA- and ethylene-biosynthesis genes are enhanced [25]. Though exogenous hormone treatment can induce fruit set and development of non-parthenocarpic fig, our understanding of the underlying mechanisms is limited.

In this study, the effect of exogenous GA_3_ treatment on the main crop of San Pedro fig was studied, and hormone contents of the female flowers and receptacle were assayed. A corresponding global transcriptome comparison demonstrated a significant number of gene ontology (GO) terms and Kyoto Encyclopedia of Genes and Genomes (KEGG) pathways with spatiotemporal regulation characteristics. The expression patterns of annotated plant hormone metabolism and signal-transduction genes were analyzed in depth. GA treatment led mainly to the regulation of auxin, ethylene and ABA biosynthesis and signaling in fig. Differential expression of plant hormone genes revealed synchronized change trends with fruit development between female flowers and receptacles following GA treatment. Our work provides new information on GA-induced fruit set in San Pedro-type fig by unraveling key pathways and genes in the expression-regulation network, which could help elucidate the molecular mechanisms underlying the diversified parthenocarpic nature of fig species.

## Methods

### Plant materials and exogenous GA treatment

A San Pedro-type fig cultivar (*Ficus carica* L. ‘Asteran’) was used in this study. The trees were planted in a commercial orchard in Beijing with regular management. Main crop fruits at pre-anthesis (15–20 mm), anthesis (20–25 mm) and post-anthesis (> 25 mm) stages were selected to evaluate the time window of GA treatment; 200 μl of 25 mg/l GA_3_ solution was injected through the ostiole into the fruit cavity with a 1-ml syringe. Solution without GA_3_ was injected into the control group. For each treatment and control group, 100 fruits were injected and fruit-set rates were calculated.

Growth curves of control and GA_3_-treated fruit were established with 30 tagged fruits using a digital slide caliper. Three biological replicates were collected from pools of at least 30 fruits 2, 4 and 10 days after treatment (DAT). For transcriptome sequencing and plant hormone analysis, main crop fruits at anthesis, 22 ± 1 mm in transverse diameter, were used. Female flowers and receptacle were carefully separated, frozen in liquid nitrogen on site, pulverized in the lab, and stored at − 80 °C for further use.

### Phytohormone content assays

The levels of IAA, GA_3_, GA_4_, zeatin and ABA were monitored by reverse-phase HPLC–MS/MS [26]. In brief, about 50 mg of the frozen and pulverized plant material was placed in 500 μl extraction solvent (isopropanol:H_2_O:concentrated HCl, 2:1:0.002, *v*/v), then shaken (100 rpm) at 4 °C for 30 min. Dichloromethane (1 ml) was added, followed by shaking (100 rpm) at 4 °C for 30 min. After centrifugation (10,000 × *g*) at 4 °C for 5 min, the solution was divided into two phases, and the lower phase (approximately 1 ml) was collected. The solvent mixture was concentrated to dryness using a Termovap sample concentrator (N-EVAP, Organomation, USA), then redissolved in 100 μl methanol. The extracted hormone solutions were run in an AB SCIEX (USA) Triple Quad™ 5500 LC–MS/MS system. For each sample, three independent and parallel extractions were carried out. Presented values are means of all replicates ± SD. Significance analysis was performed by SPSS 17.0.

### RNA extraction

Total RNA from female flowers and receptacles taken 2, 4 and 10 DAT and the corresponding controls was isolated using the CTAB method [27] and treated with RNase-free DNase I (TaKaRa, China) according to the manufacturer’s instructions. RNA quality and quantity were assessed by Nanodrop 2000 (Thermo Scientific, USA) and electrophoresis in a 1% agarose gel. RNA integrity number, analyzed by Agilent 2100 Bioanalyzer, was > 8.0.

### Library preparation and paired-end transcriptome sequencing

The mRNA of each sample’s three biological replicates were respectively enriched using cellulose oligo (dT) magnetic beads (Invitrogen, USA), then fragmented into ca. 200-bp fragments. The fragments were transcribed and double-stranded cDNA was synthesized, then end repair, 3′-end single-nucleotide A (adenine) addition and ligation of adaptors were performed according to the manufacturer’s instructions. The resultant fragments were enriched by PCR and purified using 2% Certified Low Range Ultra Agarose (Bio-Rad) to create the final cDNA library. The quantitative assay was conducted using Picogreen fluorescent dye (Invitrogen, USA) with a TBS-380 fluorimeter (Invitrogen, USA). After bridge PCR amplification on Illumina cBot using Truseq PE Cluster Kit v3-cBot-HS, paired-end (2 × 151 bp) sequencing of the cDNA library products was carried out using the Illumina HiSeq4000 platform.

### Illumina read processing and functional annotation

Clean reads were generated by removing reads with adaptors and more than 10% unknown nucleotides, and low-quality reads (the rate of reads with quality value ≤10 was more than 20%) from the raw data. Then clean reads were mapped to our reported reference sequences deposited at DDBJ/EMBL/GenBank under the accession number GDKC00000000 [25] using Bowtiealigner (http://bowtie-bio.sourceforge.net/index.shtml) [28] with no more than two mismatches allowed. The number of mapped clean reads for each transcript was normalized to FPKM (fragments per kilobase of exon model per million mapped reads) by RSEM (http://deweylab.biostat.wisc.edu/rsem/). Genes that were differentially expressed between GA_3_-treated samples and their corresponding controls were analyzed by edgeR (http://www.bioconductor.org/packages/2.12/bioc/html/edgeR.html) [29], and “false discovery rate (FDR) < 0.01 and absolute value of log_2_ (FPKM_treatment_/FPKM_control_) ≥ 1” were set as the thresholds to determine significant differences in gene expression. For GO annotation of all transcripts (http://www.geneontology.org/), Goatools (https://github.com/tanghaibao/Goatools) was used. Pathway-enrichment analysis was carried out based on the KEGG pathway database by KOBAS (http://kobas.cbi.pku.edu.cn/) [30]. Both GO and KEGG enrichment analyses were based on Fisher’s Exact Test [31] with multiple testing correction of FDR [32], and corrected *P*-value ≤0.05 was selected as the threshold for significance.

### Quantitative real-time PCR (qRT-PCR) verification

Thirteen phytohormone-related genes were selected for validation by qRT-PCR and the specific primers are shown in Additional file 1: Table S1. Isolation of total RNA was as described previously and RNase-free DNase I (D2270A, TaKaRa) treatment was performed to digest DNA in the sample. For the first-strand cDNA synthesis, 1000 ng of total RNA was reverse-transcribed with a synthesis kit (D6210A, TaKaRa). Ten-fold-diluted cDNA template was used for the qRT-PCR assay performed by the SYBR Premix Ex Taq Kit (DRR420A, TaKaRa) on a 7500 Real-Time PCR System (Applied Biosystems, USA) according to the manufacturer’s protocol. Relative quantitative method (2^−ΔΔCt^) was used to calculate the level of expression of the tested genes [33]. All of the reactions were performed in three replicates using *actin* as the internal gene [34].

## Results

### Developmental characteristics and endogenous hormone changes upon GA_3_ treatment

**Fig. 1:**
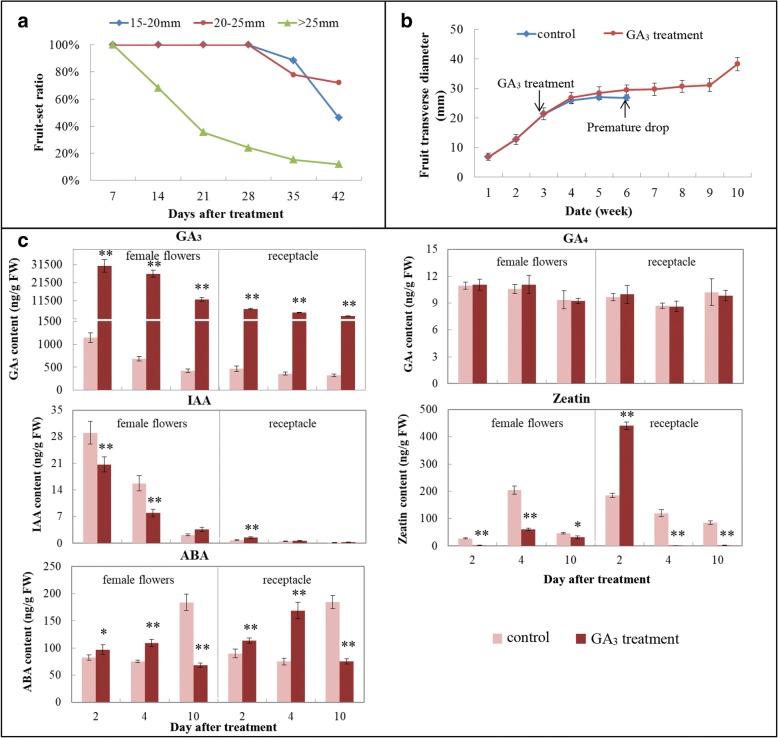
Physiological features of San Pedro-type fig main crop fruit after gibberellin (GA_3_) application. **a** Effect of application time window on fruit-set ratio. **b** Growth curves of control and GA_3_-treated fruits (GA_3_ was applied at anthesis). **c** GA_3_, GA_4_, indole acetic acid (IAA), zeatin and ABA concentration in control and GA_3_-treated female flowers and receptacle. Error bars indicate standard deviation. Significance analysis was performed by SPSS 17.0. *P ≤ 0.05, **P ≤ 0.01; FW, fresh weight

To optimize the GA_3_ treatment time window at stage I, fruits at pre-anthesis (15–20 mm in diameter), anthesis (20–25 mm) and post-anthesis (> 25 mm) stages were grouped and treated with GA_3_. GA_3_ treatment at anthesis gave a fruit-set rate of about 72% 42 DAT, much higher than the pre- and post-anthesis stage treatments (40 and 10%, respectively) (Fig. 1a). Anthesis-stage fruit with transverse diameter of 22 ± 1 mm were therefore selected for further plant hormone and transcriptome experiments. The growth curve of GA_3_-induced parthenocarpic fruit is shown in Fig. 1b; the typical double-sigmoid curve pattern was in agreement with previous reports [35]. However, fruit abscission was happened in control group at the beginning of stage II, which was called “premature drop”.

Exogenous GA_3_ treatment directly increased GA concentration in the female flowers, yielding about 26.8-, 38.8- and 28.7-fold its amount in controls 2, 4 and 10 DAT, respectively (Fig. 1c). Exogenous GA_3_ was also immediately transported into the receptacle, with 15-fold control GA_3_ levels in the treatment group 2 DAT. Decreasing trends of GA_3_ content were seen from 2 to 10 DAT in both control and GA_3_-treated female flowers and receptacles, indicating the fig tissues’ strong capacity to deplete this hormone (Fig. 1c). GA_4_ was present in fig tissues at a low and stable level, and was insensitive to GA_3_ treatment. The declining phase of native IAA in stage I female flowers was greatly accelerated by GA_3_ treatment 2 and 4 DAT, but 10 DAT, IAA degradation ceased in the treated flowers and its concentration accumulated to almost 2-fold that in the controls. At this stage, the receptacle contained much less IAA than the female flowers, and GA_3_ treatment only slightly changed its level 2 DAT (Fig. 1c); thereafter, IAA decreased to very low levels in both control and treated receptacles. Exogenous GA_3_ treatment dramatically decreased zeatin peak intensity in the female flowers 4 DAT, whereas it specifically enhanced the zeatin peak to 2.4-fold that in control receptacles 2 DAT (Fig. 1c); this action was opposite that in female flowers. ABA content was higher in GA_3_-treated female flowers and receptacles than in their control counterparts 2 and 4 DAT, whereas its level was repressed in both tissues 10 DAT, to a level that was less than half of the control. Thus, GA_3_ caused a shift in the ABA peak to 6 days earlier than its natural peak in female flowers and receptacle **(**Fig. 1c).

### Transcriptome sequencing analysis and qRT-PCR validation

The transcriptomes of female flowers and receptacles from control and GA_3_-treated fruit were sequenced 2, 4 and 10 DAT. After removing adaptors and low-quality sequences, each library generated 29.7 to 40.5 million clean reads (Additional file 1: Table S2). Sequencing quality compliance was indicated by an error ratio of single bases of less than 0.0097%; Q20 and Q30 (the percentage of bases with base quality greater than 20 or 30 among the total bases) were around 98.6 and 96.0%, respectively. The number of G and C bases was about 47.0% for each gene-expression library. Alignment of the clean reads to the reference sequence showed over 24.7 million total mapped reads, with the ratio varying from 82.11 to 83.89% of the total clean reads (Additional file 1: Table S2).

Thirteen genes related to phytohormone metabolism or signal transduction were selected for validation. Positive correlations were found between the RNA sequencing (RNA-Seq) and qRT-PCR data in all pairwise comparison groups (Additional file 1: Figure S1 and S2), confirming the consistency, validity and representativeness of our RNA-Seq data.

### Differences in gene expression pattern between control and GA_3_-treated fruit

Comparative transcriptome analysis was performed to identify the differentially expressed genes (DEGs) in GA_3_-treated samples compared to controls. Filtered by a minimum 2-fold FPKM difference with FDR < 0.01, for female flower samples, pairwise comparisons revealed 685, 294 and 1811 DEGs at 2, 4 and 10 DAT, respectively, with 400 (58.4%), 103 (35.0%) and 701 (38.7%) upregulated and 255 (41.6%), 191 (65.0%) and 1110 (61.3%) downregulated in the GA_3_-treated samples relative to controls; for receptacle samples, 908, 911 and 1672 genes were differentially expressed at 2, 4 and 10 DAT, respectively, with 436 (48.0%), 509 (55.9%) and 755 (45.2%) upregulated and 472 (52.0%), 402 (44.1%) and 917 (54.8%) downregulated in the treated samples relative to controls (Additional file 1: Figure S3).

Significantly enriched terms in the GO and KEGG databases were analyzed to identify the principle biological functions of the DEGs. In female flowers, 38, 37 and 45 significant GO terms of biological process, cellular component and molecular function were enriched 2, 4 and 10 DAT, respectively, and in the receptacle, 40, 39 and 41 GO terms were enriched (Additional file 1: Table S3). In general, the enrichment patterns of up- and downregulated genes were similar in female flowers and receptacles; the shared enriched terms in the two different tissues 2, 4 and 10 DAT were: binding (GO:0005488) and catalytic activity (GO:0003824) terms in the molecular function category, cell part (GO:0044464) and cell (GO:0005623) terms in the cellular component group, and metabolic process (GO:0008152), cellular process (GO:0009987) and single-organism process (GO:0044699) terms in the biological process group.

DEGs identified 2, 4 and 10 DAT were assigned to 4, 1 and 13 significantly different KEGG pathways in female flowers and 9, 4 and 12 pathways in the receptacle, respectively (Additional file 1: Table S4). The largest number of significantly changed pathways was found 10 DAT in both tissues. Among the hormone-related pathways, tryptophan metabolism (ko00380) was significantly enriched in female flowers 10 DAT, and plant hormone-signal transduction (ko04075) was significantly enriched in receptacles 2 and 10 DAT.

### Expression pattern of genes involved in plant hormone metabolism and signal-transduction pathways

Plant hormone-related genes differentially expressed in female flowers and/or receptacles are summarized in Additional file 1: Tables S5 and S6. Briefly, most of the genes belonged to the ethylene-metabolism and response pathways, followed by auxin, ABA and GA; relatively fewer DEGs were related to cytokinin. A FPKM value > 10 for at least one sample was set as the threshold to select genes for further analysis. The expression profiles of the rest of the hormone-related DEGs, with FPKM values of all samples < 10, are shown in Additional file 1: Figure S4.

### Gibberellin

**Fig. 2:**
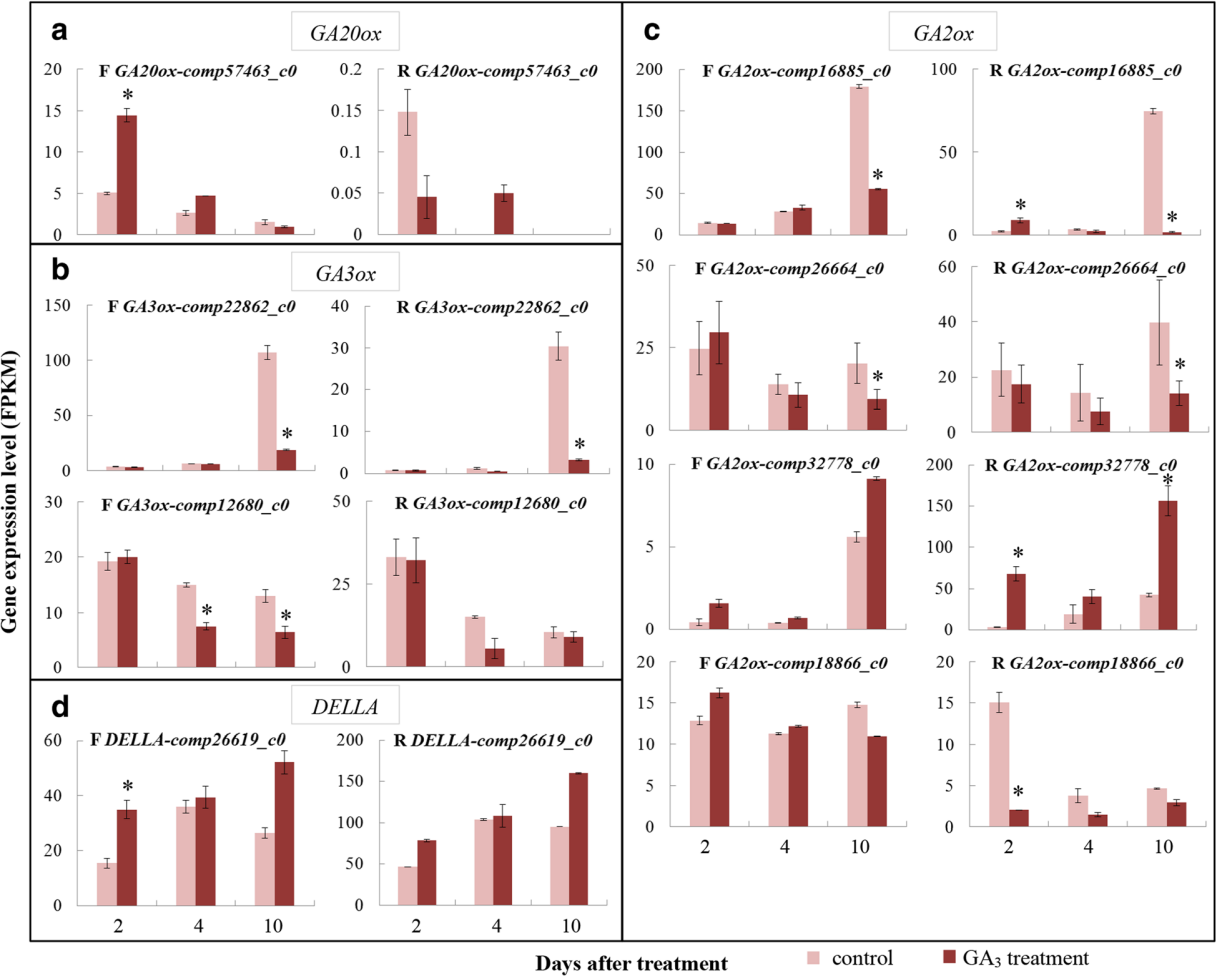
Expression patterns of GA-metabolism and signaling genes. **a** *GA20ox*. **b** *GA3ox*. **c** *GA2ox*. **d** *DELLA*. F, female flowers; R, receptacle. *Fold change of FPKM ≥2 and FDR ≤ 0.001

We identified 10 GA biosynthesis and catabolism genes encoding GA 20-oxidase (GA20ox), GA 3-oxidase (GA3ox) and GA 2-oxidase (GA2ox) (Additional file 1: Table S5). The expression level of the only *GA20ox* identified in this study was dramatically higher in female flowers than receptacles, and it was significantly upregulated in GA_3_-treated female flowers 2 DAT compared to controls, then downregulated until 10 DAT in both control and treated samples (Fig. 2a). Two *GA3ox* genes showed different expression patterns: *comp22862_c0* was weakly expressed in all studied samples 2 and 4 DAT and showed sharp upregulation in control female flowers and receptacle 10 DAT, to significantly higher levels compared to their GA_3_-treated counterparts; *comp12680_c0* showed the highest expression level 2 DAT and then a decrease during development, and was generally downregulated in the two different tissues after GA_3_ treatment (Fig. 2b).

Among all *GA2ox* genes, *comp16885_c0* was most highly expressed, but its expression was repressed 3.3- and 41.1-fold in GA_3_-treated female flowers and receptacle compared to controls at 10 DAT, respectively. Another *GA2ox* gene (*comp26664_c0*) also exhibited significantly decreased expression in GA_3_-treated vs. control samples at 10 DAT. However, *comp32778_c0* expression was upregulated after treatment at all sampling stages in both female flowers and receptacle, and remarkably higher expression was found in the receptacle vs. female flowers. In addition, *comp18866_c0* only showed a significant difference in expression between control and treated receptacles 2 DAT, with the latter being 7.5-fold lower than the former (Fig. 2c).

With respect to GA-signal transduction, only one DEG encoding DELLA protein was identified, which was more highly expressed in GA_3_-treated fruit than in controls; its upregulation was significant in GA_3_-treated female flowers 2 DAT (Fig. 2d). These results suggest that exogenous GA_3_ treatment mainly influences GA-biosynthesis and catabolism pathways in both female flowers and receptacle, but not the signal-transduction process.

### Auxin

**Fig. 3:**
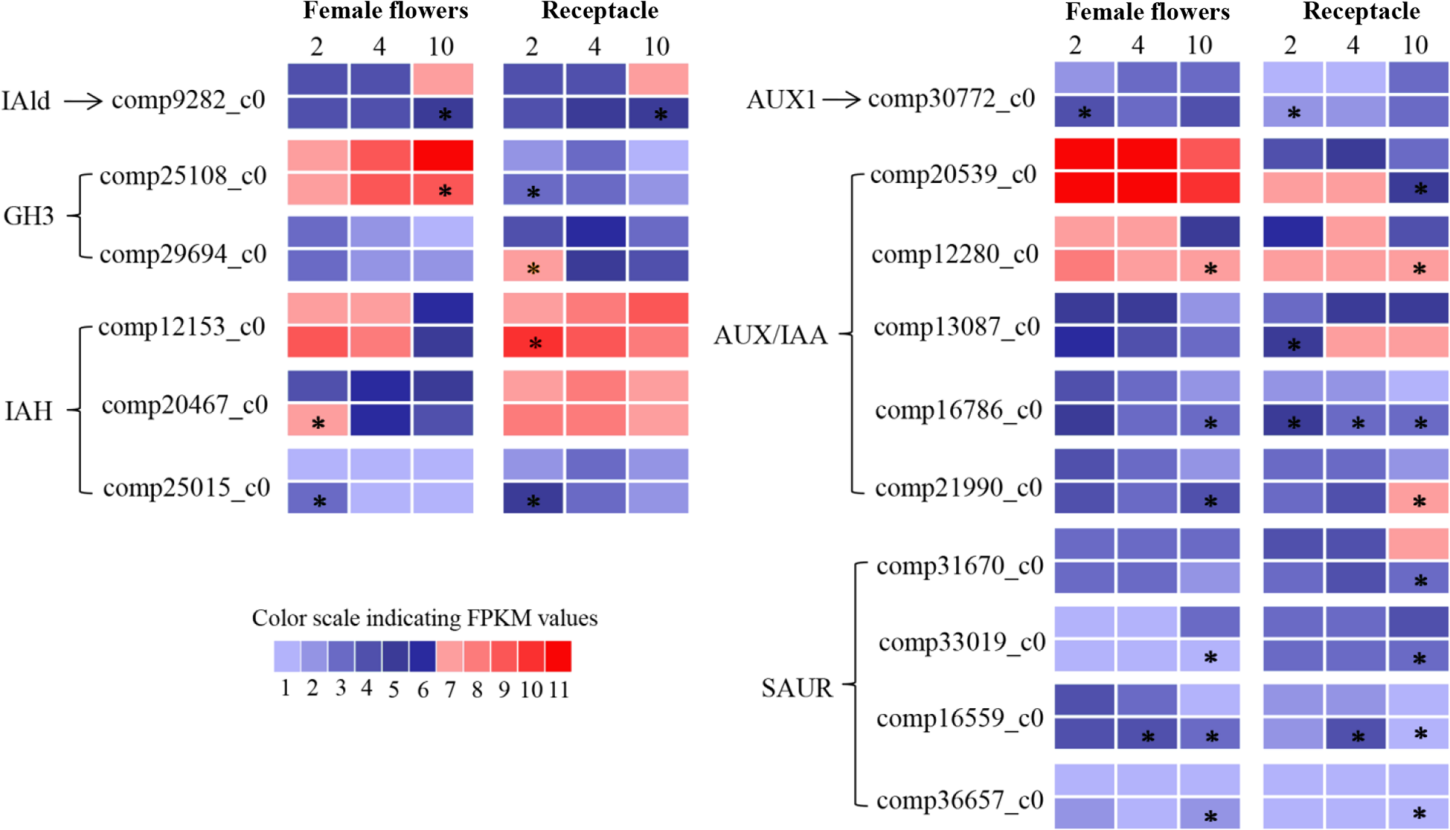
Expression patterns of auxin-metabolism and signaling genes. Grids with 11 different colors represent FPKM values of 0–10, 10–20, 20–40, 40–60, 60–80, 80–100, 100–200, 200–300, 300–400, 400–500, and over 500, respectively. For each gene, the upper and lower rows refer to the results of control and GA_3_ treatment, respectively. 2, 4 and 10 = days after treatment. *Fold change of FPKM ≥2 and FDR ≤ 0.001

In GA_3_-treated fig, auxin-pathway genes constituted the largest group of DEGs among growth- and development-promoting phytohormones (Additional file 1: Table S5). The expression level of *indole-3-acetaldehyde dehydrogenase* (*IAld*) remained stable in female flowers and receptacle 2 and 4 DAT, whereas 10 DAT, it was upregulated in the two control tissues, to significantly higher levels than in their GA_3_-treated counterparts (Fig. 3). Two auxin-responsive *Gretchen Hagen 3* (*GH3*) family genes revealed different expression patterns in the female flowers and similar patterns in the receptacles. In female flowers, *comp25108_c0* showed continuously increasing expression during development in control tissues, more than 2-fold higher than in the treated tissues 10 DAT; in contrast, the other *GH3* (*comp29694_c0*) decreased from 2 to 10 DAT in female flowers, and showed no significant difference between control and treated flowers. In receptacles, both *GH3* genes exhibited over 2-fold upregulation 2 DAT (Fig. 3). Three *IAA-amino acid hydrolase* (*IAH*) genes demonstrated overall moderate changes in expression during development—increasing from 2 to 4 DAT and then decreasing in control female flowers and receptacle; however, GA_3_ treatment significantly elevated *IAH* expression 2 DAT, followed by a continuous decrease, resulting in similar expression levels between control and treated tissues 4 and 10 DAT. This implies that GA only transiently modulates *IAH* expression in female flowers and receptacles (Fig. 3).

With respect to the auxin-response pathway (Additional file 1: Table S5), *auxin-influx carrier* (*AUX1*) demonstrated higher expression in GA_3_-treated fruit female flowers than in controls on all sampling days, and in the treated receptacle, upregulation was observed 2 DAT, whereas downregulation was identified 10 DAT (Fig. 3). Expression patterns of four *small auxin-up RNA* (*SAUR*) genes could be separated into two groups: DEGs *comp31670_c0* and *comp33019_c0* were significantly repressed in GA_3_-treated fruit female flowers and receptacle 10 DAT, whereas *comp16559_c0* and *comp36657_c0* were generally upregulated in the treatment group on all three sampling days relative to their control counterparts (Fig. 3). The negative auxin-response regulator *AUX/IAA* revealed divergent expression patterns between female flowers and receptacle and between control and treatment groups. In general, *AUX/IAA* presented higher expression in GA_3_-treated samples than in controls (Fig. 3).

### Cytokinin

**Fig. 4:**
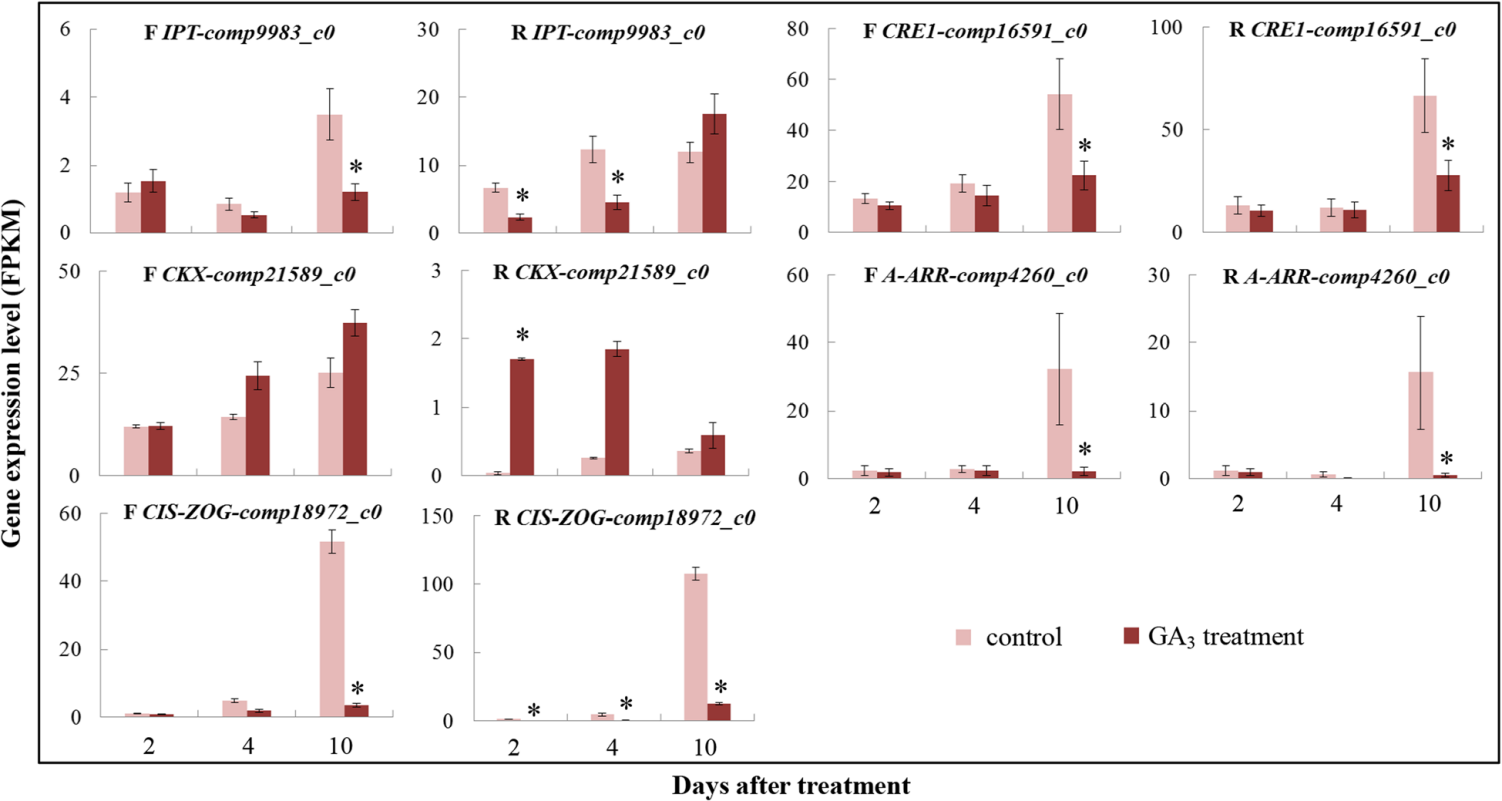
Expression patterns of cytokinin-metabolism and signaling genes. F, female flowers; R, receptacle. *Fold change of FPKM ≥2 and FDR ≤ 0.001

Differential expression of cytokinin metabolism and signal-transduction genes was identified (Fig. 4, Additional file 1: Table S5). *Isopentenyl transferase* (*IPT*) expression was higher in GA_3_-treated female flowers than controls 2 DAT, but lower 4 and 10 DAT. On the other hand, in the receptacle, *IPT* was downregulated 2 and 4 DAT, and moderately upregulated 10 DAT. Compared to controls, *cytokinin oxidase/dehydrogenase* (*CKX*) was upregulated in GA_3_-treated samples, and its expression was remarkably higher in female flowers vs. receptacle. *Cis-zeatin O-glucosyltransferase* (*CIS-ZOG*) showed very low expression in all samples 2 and 4 DAT, and maintained this low expression in GA_3_-treated fruit, whereas 10 DAT, it was 14.7- and 8.6-fold higher in control vs. GA_3_-treated female flowers and receptacle, respectively (Fig. 4). Note that in rice, the activity of cis-zeatin is comparable to that of trans-zeatin and can upregulate cytokinin-inducible genes, whereas overexpression of cis-ZOG delays rice leaf senescence [36].

In cytokinin-signal transduction, the cytokinin receptor *cytokinin response*1 (*CRE1*) was downregulated in both female flowers and receptacle after GA_3_ treatment, especially 10 DAT (Fig. 4). The negative *A-type Arabidopsis response regulators* (*ARR*s) remained stable with a low expression level in GA_3_-treated fruit at all tested stages, whereas a very significant increase was identified in controls 10 DAT, presenting 16- and 31-fold upregulation in female flowers and receptacle, respectively, compared to the corresponding GA_3_-treated tissues (Fig. 4).

### ABA

**Fig. 5:**
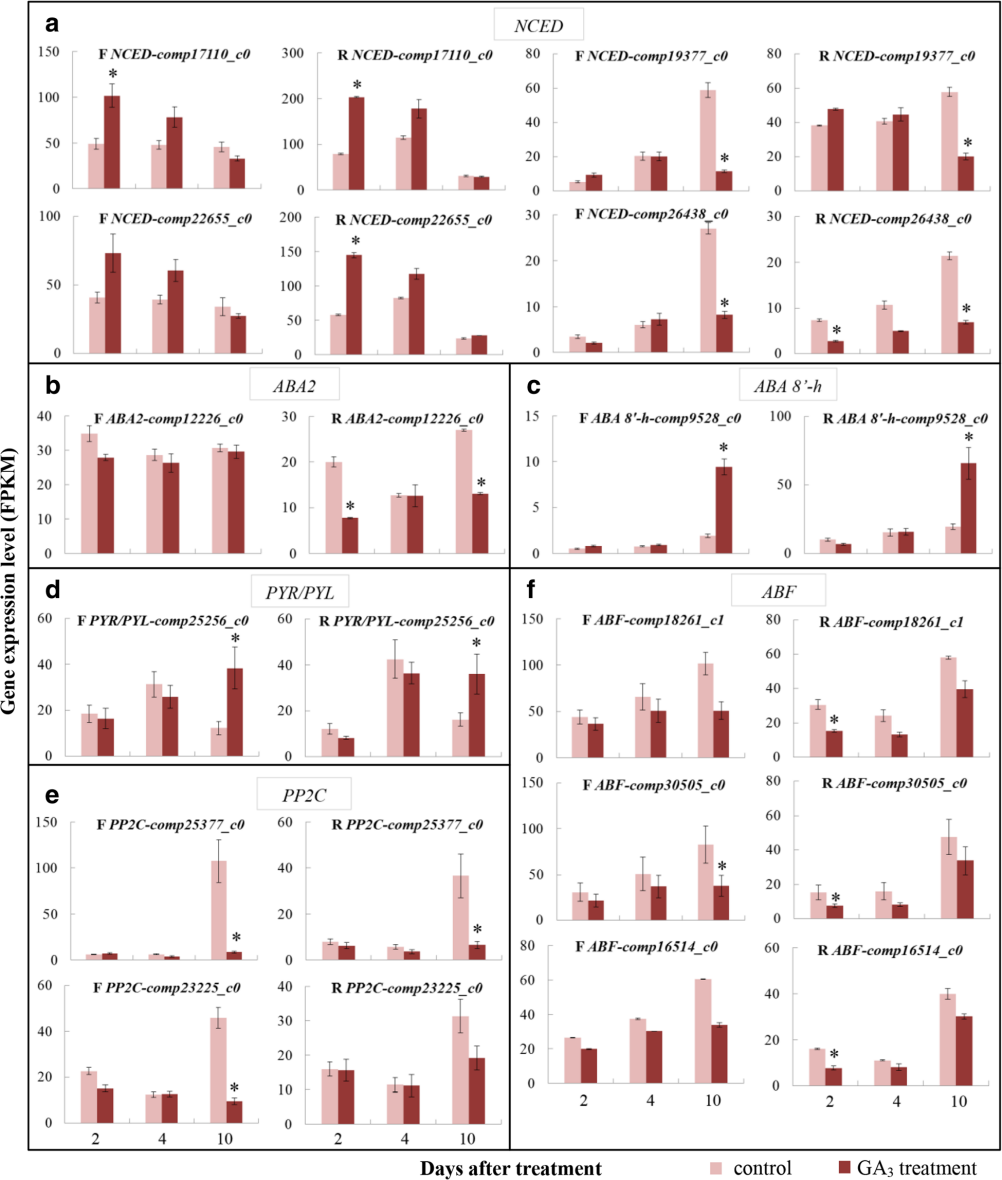
Expression patterns of ABA-metabolism and signaling genes. **a** *NCED*. **b** *ABA2*. **c** *ABA 8′-h*. **d** *PYR/PYL*. **e** *PP2C*. **f** *ABF*. F, female flowers; R, receptacle. *Fold change of FPKM ≥2 and FDR ≤ 0.001

All identified ABA-synthesis, catabolism and response DEGs are shown in Additional file 1: Table S6. NECD (9-cisepoxycarotenoid dioxygenase) is the first committed and rate-limiting enzyme in ABA biosynthesis [37]. Two *NCED* genes—*comp17110_c0* and *comp22655_c0*—were enhanced in the both tissues 2 and 4 DAT with GA_3_ (Fig. 5a). In controls, their expression was relatively stable during development in female flowers, and rose first and then fell at the 10 DAT in the receptacle; however, decreasing trends were found in both female flowers and receptacle of GA_3_-treated fruit. The other two *NCED*—*comp19377_c0* and *comp26438_c0*—generally increased in the two tissues of control fruit during development, especially from 4 to 10 DAT, whereas their levels changed moderately in GA_3_-treated tissues; ultimately, their expression was significantly lower in treatment vs. control tissues 10 DAT (Fig. 5a). *ABA2*, encoding another ABA-biosynthesis enzyme, was downregulated in GA_3_-treated vs. control tissues, but with no significant difference in the female flowers (Fig. 5b). The process catalyzed by ABA 8′-hydroxylase (ABA 8′-h) is considered to be the main pathway for ABA catabolism [37]. *ABA 8′-h* expression remained relatively stable in the controls, whereas remarkable upregulation was found in the GA_3_-treated samples 10 DAT—4.9- and 3.3-fold higher in female flowers and receptacle, respectively, than in controls (Fig. 5c).

In the ABA-signaling pathway, *PYR/PYL* expression was slightly lower in GA_3_-treated samples than in controls 2 and 4 DAT, but significantly higher 10 DAT (Fig. 5d). Protein phosphatase 2C (PP2C) usually represses ABA responses. *PP2C* expression was not significantly different between the control and treated fruit 2 and 4 DAT, whereas it was dramatically downregulated in GA_3_-treated female flowers and receptacle 10 DAT (Fig. 5e). ABRE binding factor (ABF) functions as a positive regulator of ABA signaling. The *ABF* genes’ expression was consistent with an increasing tendency from 2 to 10 DAT in both control and GA_3_-treated samples, and their expression was more or less downregulated in all studied samples after GA_3_ treatment (Fig. 5f).

### Ethylene

**Fig. 6:**
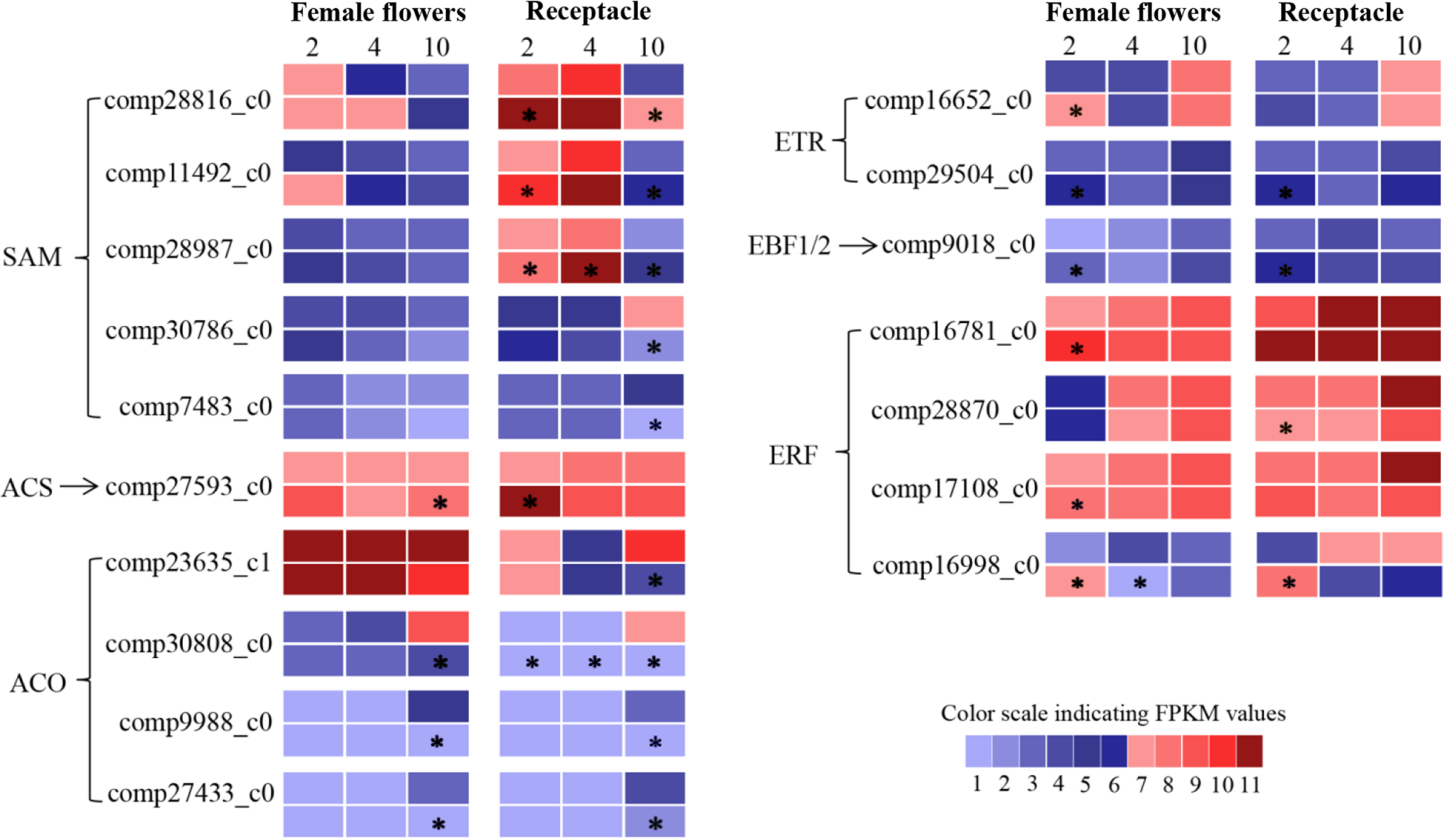
Expression patterns of ethylene-biosynthesis and signaling genes. Grids with 11 different colors represent FPKM values 0–10, 10–20, 20–40, 40–60, 60–80, 80–100, 100–200, 200–300, 300–400, 400–500 and over 500, respectively. For each gene, upper and lower rows refer to the results of control and GA_3_ treatment, respectively. 2, 4 and 10 = days after treatment. *Fold change of FPKM ≥2 and FDR ≤ 0.001

A large number of ethylene biosynthesis- and signaling-related DEGs were found in female flowers and/or receptacles after GA_3_ treatment (Additional file 1: Table S6). *S-adenosylmethionine synthetase* (*SAM*) displayed the highest expression 2 DAT in control and GA_3_-treated female flowers, followed by a gradual decrease, with no significant differences between control and treatment (Fig. 6). Three *SAM* genes—*comp28816_c0*, *comp11492_c0* and *comp28987_c0*—peaked 4 DAT and showed their lowest expression 10 DAT in control and GA_3_-treated receptacles; they demonstrated higher expression after treatment than in controls. The other two *SAM* genes—*comp30786_c0* and *comp7483_c0*—increased during the control samples’ development, but showed decreasing trends after GA_3_ treatment, leading to 6.8- and 7.7-fold downregulation 10 DAT, respectively, in the GA_3_-treated receptacle (Fig. 6). One gene annotated as *1-aminocyclopropane-1-carboxylate* (*ACC*) *synthase* (*ACS*) presented higher expression in GA_3_-treated samples than in controls. *ACC oxidase* (*ACO*) genes, except *comp23635_c1*, had low expression in all tested control and GA_3_-treated samples 2 and 4 DAT, whereas dramatic upregulation was detected in control fruit female flowers and receptacle 10 DAT, resulting in significantly higher expression in the two control vs. treated tissues (2.5- to 42-fold) (Fig. 6).

In the ethylene-response pathway, *ETR* transcripts increased during development, showing the highest expression levels 10 DAT in controls. For GA_3_-treated samples, their expression levels fell first and then rose, with a peak at 2 or 10 DAT (Fig. 6). The expression levels of *ETR* and *EBF1/2* were significantly upregulated in GA_3_-treated female flowers and receptacle 2 DAT. In general, *ERF* transcripts revealed an increasing trend in control fruit female flowers and receptacle, whereas they presented divergent expression patterns after GA_3_ treatment: *comp16781_c0* and *comp16998_c0* had the highest expression 2 DAT in female flowers and receptacle; *comp28870_c0* and *comp17108_c0* displayed upward trends during development similar to their expression in the controls (Fig. 6). *ERF*s were generally upregulated in the two treatment tissues 2 DAT, whereas 10 DAT, their expression levels were slightly lower in GA_3_-treated vs. control receptacles.

## Discussion

Our aim was to investigate the role of GA in parthenocarpy induction in fig by comparing control and GA_3_-treated fruit’s female flowers and receptacles. We focused our study on 2, 4 and 10 days after exogenous GA_3_ application in a San Pedro-type fig cultivar’s main crop, which is genetically non-parthenocarpic. By Illumina RNA-Seq transcriptome analysis, 1.3- and 3.1-fold more DEGs were identified in the receptacle vs. female flowers at 2 and 4 DAT, respectively, whereas the number of DEGs was a little higher in female flowers than in the receptacle 10 DAT. This implied that aside from the reproductive female flowers, which mainly influence fruit development [38], the vegetative receptacle tissue might also have some regulatory effect on parthenocarpic fruit set and growth. GA, auxin, cytokinin, ABA and ethylene metabolism- and response-related genes showed differential expression between control and GA_3_-treated fruit, and phytohormone content assay revealed a new balance of endogenous phytohormone levels in both female flowers and receptacle following exogenous GA treatment (Fig. 1c). This indicated that parthenocarpy induction by GA in fig is the result of the coordinated action of different phytohormones.

**Fig. 7:**
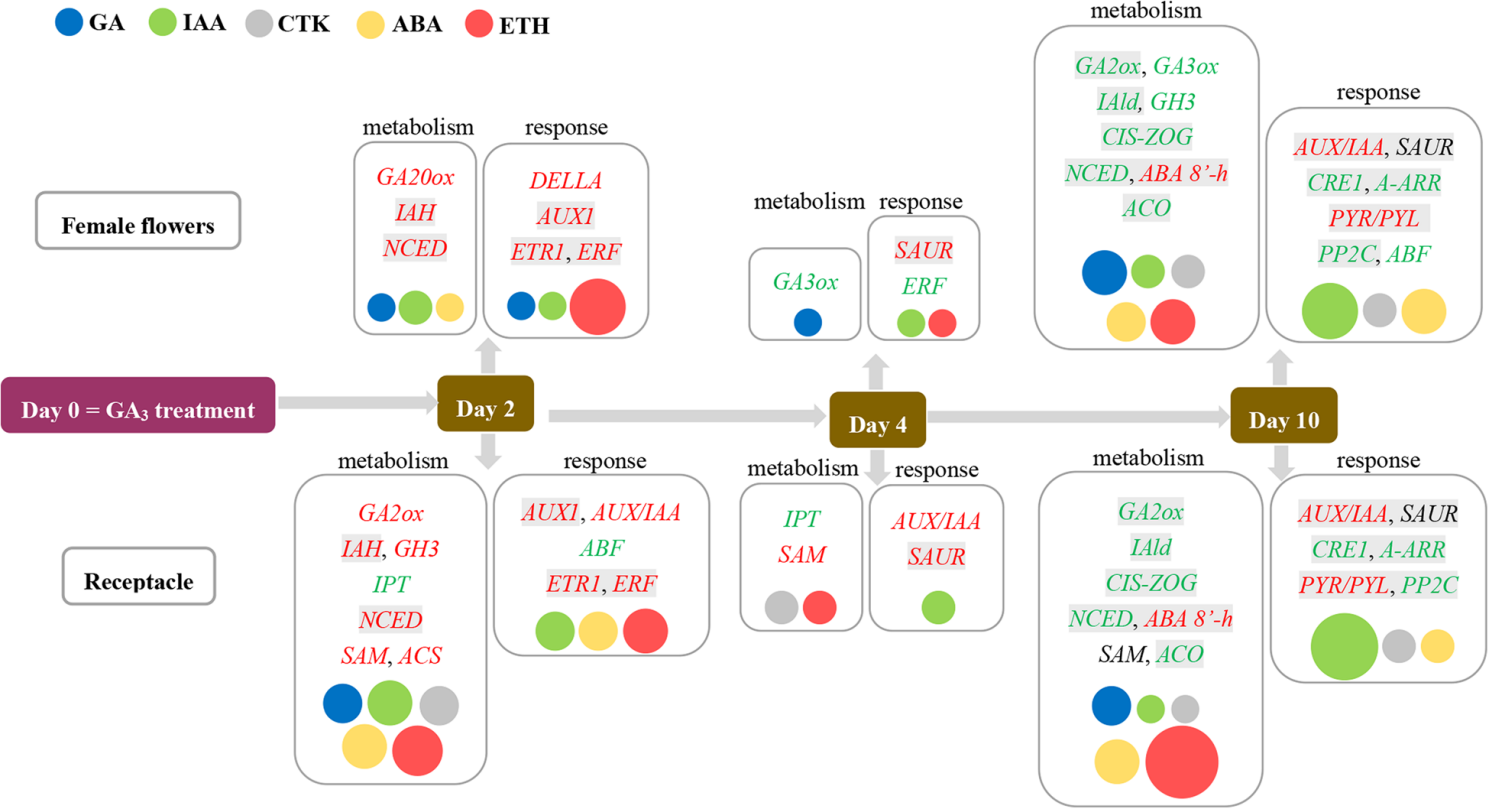
Summary of main differentially expressed phytohormone-related genes identified in female flowers and receptacle after GA_3_ treatment. Different colored circles represent different phytohormones and circle size indicates relative number of DEGs—the higher the number, the bigger the circle. Genes in red (green) color are upregulated (downregulated). Gray highlight represents genes with same changing trend in both female flowers and receptacle following GA_3_ application

Exogenous GA_3_ application reset the transcriptional expression of plant hormone-related genes. In female flowers, 4, 1 and 15 phytohormone-metabolism genes and 8, 2 and 12 response-related genes were significantly differently expressed 2, 4 and 10 DAT, respectively. In the receptacle, 19, 4 and 18 phytohormone-metabolism and 10, 2 and 12 signal-transduction DEGs were identified 2, 4 and 10 DAT, respectively. The higher representation of plant hormone transcripts’ differential expression in receptacles compared to female flowers may further suggest an indelible role for receptacle tissue in controlling fruit set and development. Concrete spatiotemporal transcriptional changes in phytohormone-related genes induced by GA_3_ are summarized in Fig. 7. There were more common DEGs with the same changing trends in female flowers and receptacles 10 DAT than 2 and 4 DAT, indicating that GA_3_ treatment induces synchronized patterns of changing gene expression between the two tissues as fruit development progresses. In addition, in both female flowers and receptacles 10 DAT, the number of DEGs related to ethylene metabolism was highest, followed by those related to GA and ABA metabolism; for phytohormone response, the highest number of DEGs were related to auxin.

### Phytohormone-metabolism response to exogenous GA_3_

Previous studies have shown that transcription of GA-metabolism genes is regulated by the content of bioactive GA [2, 39]. GA oxidases modulate the GA-biosynthetic pathway mainly through feedback-loop mechanisms [40] and expression regulation that depends on the tissue and developmental stage [39, 41]. Exogenous GA application before or after bloom in grapevine leads to downregulation of the GA-biosynthetic genes *GA20ox* and *GA3ox* and upregulation of the GA-catabolic gene *GA2ox* [15, 42, 43]; this has also been observed in *Arabidopsis* [39] and tobacco [44]. Conversely, in the present study, *GA20ox* was upregulated in female flowers 2 DAT and *GA2ox* was repressed in both female flowers and receptacle 10 DAT. This difference could be explained by the change in endogenous GA content: in both fig and grapevine, GA content increases significantly and immediately after treatment and then decreases; however, for grapevine, GA content drops to levels equal to or significantly less than the control 3 DAT [43, 45], whereas in fig, it is still significantly higher than the control 10 DAT (Fig. 1c).

Auxin is one of the main regulators of fruit set and development. Similar to [42], auxin-biosynthesis genes, e.g. *YUCCA*, did not show significant regulation after GA_3_ treatment in fig. The main differentially expressed auxin-metabolism genes identified in the present study were *IAH* and *GH3*. The former encodes enzymes that produce free IAA from IAA–amino acid conjugates, whereas the latter catalyzes auxin conjugation [46]. *IAH* and *GH3* were mainly significantly upregulated 2 DAT; upregulation of *GH3* has also been reported previously [42], indicating that bioactive auxin levels are co-mediated by *IAH* and *GH3* in fig. Cytokinin induces parthenocarpy by altering the expression of GA-biosynthesis genes [9, 16]. Only a few metabolic genes’ transcriptional levels were regulated by GA_3_ treatment in our dataset. For female flowers, cytokinin-metabolism transcripts were only mediated by GA_3_ treatment 10 DAT, whereas for the receptacle, exogenous GA_3_ also regulated cytokinin metabolism 2 and 4 DAT (Fig. 7). Zeatin concentration increased dramatically in the receptacle 2 DAT, then decreased to a markedly lower level than controls, suggesting that cytokinin-biosynthesis genes respond more rapidly (in under 2 days) in the receptacle following GA_3_ application.

Aside from growth-promoting hormones, GA_3_ treatment also modulated the biosynthesis of ABA and ethylene, which mainly play GA-antagonistic roles in the control of many plant developmental processes [47]. Enhancement of ABA and ethylene biosynthesis 2 DAT might reflect ‘feedback’ regulation of the highly significantly increased GA_3_ content in fig fruit following exogenous GA_3_ application. This ‘feedback’ response then declines, with almost no ABA- or ethylene-related DEGs being identified 4 DAT. Along with fruit development, both female flowers and receptacle revealed suppression of ABA and ethylene biosynthesis 10 DAT, with downregulation of *NCED* and *ACO* and upregulation of *ABA 8′-h*, in accordance with the lower ABA level in GA_3_-treated fruits*.* Previous reports have shown that expression of the ethylene-biosynthesis genes *ACO*s decreases during parthenocarpic fruit set and development or after GA_3_ treatment [13].

### Phytohormone signaling in response to exogenous GA_3_

DELLA protein has been characterized as a negative regulator in GA signaling [48]. One *DELLA* gene exhibited upregulation after GA_3_ application, most markedly in female flowers 2 DAT, in accordance with previously reported results [7, 13, 43]. This indicates GA-perception feedback regulation in female flowers and receptacle.

Studies of the crosstalk between GA and auxin have revealed that GA-induced parthenocarpy arises from the interaction of GA with auxin signaling [42], and that auxin-induced fruit set is mediated in part by GA [9]. In the present study, *AUX/IAA* gene expression was significantly upregulated in the receptacle on all sampling days and in female flowers 10 DAT, suggesting feedback regulation of the auxin response following GA_3_ treatment, in agreement with results in grape [15]. In addition to the *AUX/IAA*s, differential expression of *SAUR* transcript levels was seen in both female flowers and receptacles 10 DAT (Fig. 3). Further study is needed to elucidate their roles in fig fruit development. DEGs related to cytokinin signaling were only identified 10 DAT in the female flowers and receptacle (Fig. 7).

GA_3_ treatment also changed the signaling of ABA and ethylene. Enhancement of ethylene signaling was identified 2 DAT. Crosstalk internodes between GA and the ethylene-signaling pathway were elucidated by the role of group VII ERFs as DELLA partners [49], and ERF11 activates GA biosynthesis and signaling [50]. Genes encoding ABA receptor PYR/PYL and PP2C, a negative regulator in the ABA-response pathway, displayed upregulation and downregulation, respectively, in both tissues 10 DAT, leading to enhancement of ABA-signal transduction in both female flowers and receptacle. A recent study on rice revealed the central role of PYR/PYL in the antagonistic action of GA and ABA [51].

## Conclusions

GA_3_ treatment, performed to induce parthenocarpy in San Pedro-type fig main crop, resulted in a highly significant GA_3_ content increment in both female flowers and receptacle tissues of fig fruit. Changes in the expression of key genes in plant hormone-synthesis and metabolism pathways—as reflected by modulated endogenous plant hormone levels—and in the hormone-signaling pathway, converted the pre-dropping fruit pattern to an artificial parthenocarpic developmental pattern. Spatiotemporal characteristics of the changes in plant hormone levels and gene-expression patterns in GA_3_-induced fig parthenocarpy were reflected by differentially reacting female flowers and receptacle tissue, and temporal waves in major plant hormone and relevant genes’ expression were illustrated. Further study on fig fruit development with the application of other exogenous plant hormones, e.g., auxin and cytokinin, will serve to further understand the mechanisms of GA-induced parthenocarpy and the key plant hormones in fig fruit set and development.

## Additional file

Additional file 1: **Table S1.** Primer sequences of genes used for validation of RNA-Seq results by quantitative real-time PCR, **Table S2.** Summary of sequencing results for control and GA_3_-treated female flowers and receptacles, **Table S3.** Significant GO terms (corrected *P*-value ≤0.05) identified between control and GA_3_-treated female flowers and receptacles, **Table S4.** Significant KEGG pathways (corrected *P*-value ≤0.05) identified between control and GA_3_-treated female flowers and receptacles, **Table S5.** All gibberellin-, auxin- and cytokinin-synthesis, catabolism and response transcripts identified in this study which were differentially expressed (FDR < 0.01 and the absolute value of log_2_ (FPKM_treatment_/FPKM_control_) ≥ 1) in at least one pairwise comparison group, **Table S6.** All abscisic acid- and ethylene-synthesis, catabolism and response transcripts identified in this study which were differentially expressed (FDR < 0.01 and the absolute value of log_2_ (FPKM_treatment_/FPKM_control_) ≥ 1) in at least one pairwise comparison group, **Figure S1.** Verification of RNA-Seq results by qRT-PCR. Bars represent standard deviation. F, female flowers; R, receptacle, **Figure S2.** Correlation of fold changes in gene expression between RNA-Seq and qRT-PCR. Equation of linear regression and correlation coefficient (R^2^) are shown. DAT, days after treatment, **Figure S3.** Number of differentially expressed genes (FDR ≤ 0.001 and log_2_ (FPKM_treatment_/FPKM_control_) ≥ 1 or ≤ − 1) between control and GA_3_-treated female flowers and receptacles. DAT, days after treatment, **Figure S4.** Heat maps of hormone-related genes with low expression. FPKM of all samples was < 10. (PDF 1241 kb)

## Abbreviations

ABA 8′-h: ABA 8′-hydroxylase
ABA: Abscisic acid
ABF: ABRE binding factor
ACC: 1-aminocyclopropane-1-carboxylate
ACS: ACC synthase; ACO: ACC oxidase
ARRs: *Arabidopsis* response regulators
AUX1: auxin-influx carrier
CIS-ZOG: cis-zeatin O-glucosyltransferase
CKX: Cytokinin oxidase/dehydrogenase
CRE1: Cytokinin response1
DAT: Days after treatment
DEGs: Differentially expressed genes
FDR: False discovery rate
FPKM: Fragments per kilobase of exon model per million mapped reads
GA20ox: GA 20-oxidase
GA2ox: GA 2-oxidase
GA3ox: GA 3-oxidase
GAs: Gibberellins
GH3: Gretchen Hagen 3
GO: Gene Ontology
IAA: Indole-3-acetic acid
IAH: IAA-amino acid hydrolase
IAld: Indole-3-acetaldehyde dehydrogenase
IPT: Isopentenyl transferase
KEGG: Kyoto Encyclopedia of Genes and Genomes
NECD: 9-cisepoxycarotenoid dioxygenase
PP2C: Protein phosphatase 2C
qRT-PCR: Quantitative real-time PCR
RNA-Seq: RNA sequencing
SAM: S-adenosylmethionine synthetase
SAUR: Small auxin-up RNA
