# Supplementary material for: Transcriptome analysis unravels spatiotemporal modulation of phytohormone-pathway expression underlying gibberellin-induced parthenocarpic fruit set in San Pedro-type fig (Ficus carica L.)
Source: BMC Plant Biol. 2018 Jun 1;18:100. doi: 10.1186/s12870-018-1318-1 (PMC5984833; doi:10.1186/s12870-018-1318-1)
Supplement: Supplementary file 1 — Table S1. Primer sequences of genes used for validation of RNA-Seq results by quantitative real-time PCR, Table S2. Summary of sequencing results for control and GA3-treated female flowers and receptacles, Table S3. Significant GO terms (corrected P-value ≤0.05) identified between control and GA3-treated female flowers and receptacles, Table S4. Significant KEGG pathways (corrected P-value ≤0.05) identified between control and GA3-treated female flowers and receptacles, Table S5. All gibberellin-, auxin- and cytokinin-synthesis, catabolism and response transcripts identified in this study which were differentially expressed (FDR < 0.01 and the absolute value of log2 (FPKMtreatment/FPKMcontrol) ≥ 1) in at least one pairwise comparison group, Table S6. All abscisic acid- and ethylene-synthesis, catabolism and response transcripts identified in this study which were differentially expressed (FDR < 0.01 and the absolute value of log2 (FPKMtreatment/FPKMcontrol) ≥ 1) in at least one pairwise comparison group, Figure S1. Verification of RNA-Seq results by qRT-PCR. Bars represent standard deviation. F, female flowers; R, receptacle, Figure S2. Correlation of fold changes in gene expression between RNA-Seq and qRT-PCR. Equation of linear regression and correlation coefficient (R2) are shown. DAT, days after treatment, Figure S3. Number of differentially expressed genes (FDR ≤ 0.001 and log2 (FPKMtreatment/FPKMcontrol) ≥ 1 or ≤ − 1) between control and GA3-treated female flowers and receptacles. DAT, days after treatment, Figure S4. Heat maps of hormone-related genes with low expression. FPKM of all samples was < 10. (PDF 1241 kb) [file 12870_2018_1318_MOESM1_ESM.pdf]

Transcriptome analysis unravels spatiotemporal modulation of phytohormone-pathway expression underlying gibberellin-induced parthenocarpic fruit set in San Pedro-type fig (*Ficus carica* L.)

Lijuan Chai[1†], Peng Chai[1†], Shangwu Chen[2], Moshe A. Flaishman[3], Huiqin Ma[1*]

[1]College of Horticulture, China Agricultural University, Beijing, P.R. China

[2]College of Beijing Laboratory for Food Quality and Safety, College of Food Science and Nutritional Engineering, China Agricultural University, Beijing, P.R. China

[3]Department of Fruit Tree Sciences, Agricultural Research Organization, Volcani Center, Bet-Dagan, Israel

Lijuan Chai: clj2010@cau.edu.cn

Peng Chai: chaipeng@cau.edu.cn

Shangwu Chen: swchen@cau.edu.cn

Moshe A. Flaishman: vhmoshea@volcani.agri.gov.il

Huiqin Ma: hqma@cau.edu.cn

*Correspondence: Huiqin Ma, hqma@cau.edu.cn

[†]These authors contributed equally to this work

Table S1 Primer sequences of genes used for validation of RNA-Seq results by quantitative real-time PCR

Gene ID	Annotation in NR database	Forward primer (5'→3')	Reverse primer (5'→3')
comp12280_c0	gi:587835799/auxin-induced protein IAA4 [*Morus notabilis*]	CGGATCTGCCACACTTACTGCT	ACGCTGATCACGAGGACCAC
comp16786_c0	gi:587939152/auxin-induced protein AUX22 [*Morus notabilis*]	AGCATCCAGTCTCCGTCT	ATTCGGATCTTGCAATGGCTT
comp29694_c0	gi:587887925/hypothetical protein L484_011493 [*Morus notabilis*]	ACCTTTGTCCAGCCCCGGAAC	CCGACCGCGAAACCTACAAGTCCA
comp57463_c0	gi:587886862/gibberellin 20 oxidase 3 [*Morus notabilis*]	CCCATTCGACAATGCCACAAAGGT	CCAGAACAACAAGTGGAACTCCGT
comp12680_c0	gi:587893059/gibberellin 3-beta-dioxygenase 4 [*Morus notabilis*]	CGCCAATTTTCGCCCTATGTCC	CACCGTCAGCAGTCAACCCTC
comp16885_c0	gi:587973425/gibberellin 2-beta-dioxygenase 1 [*Morus notabilis*]	ACATTCCTCGGCTGAATCGTC	GGGTCGAATACCTCCTCTTAACCAC
comp17110_c0	gi:587942803/hypothetical protein L484_021647 [*Morus notabilis*]	GCAAAATCCCGAACCGCGACT	TTCCGACGATGATGCACGACT
comp19377_c0	gi:587862256/hypothetical protein L484_024617 [*Morus notabilis*]	CCCTCCAGAGTCCCATACGGTT	ATCTACAAGCCTCGCAGACACGAT
comp23225_c0	gi:587870483/putative protein phosphatase 2C 6 [*Morus notabilis*]	ACATCCCAAAGCCCATCACTTGC	AGGCCATGGATAATTCCCGTTCCAG
comp25377_c0	gi:587848632/protein phosphatase 2C 16 [*Morus notabilis*]	CCCTTTTGTTCATGTAGGTGCTT	CCTTCTCCATCAACTCGCGTTC

comp18261_c1	gi:375155309/ABA responsive element-binding protein [*Solanum torvum*]	GCACCGTTTCTTCGCTCCC	ACAAGGCTTATACAATGGAACTGGA
comp11492_c0	gi:255556894/s-adenosylmethionine synthetase, putative [*Ricinus communis*]	ACACATCTCTTATTGCGGCCTC	TGTTAAGATCCTCAAGCCGAAGGT
comp9988_c0	gi:587870860/1-aminocyclopropane-1-carboxylate oxidase 5 [*Morus notabilis*]	ACATCTTCCCAGTCAGCGTTGTCC	ACTTCAACAACTCCAAAGCCGTCA

Table S2 Summary of sequencing results for control and GA_3-treated female flowers and receptacles

Sample	Total clean reads	Total base pairs	Total mapped reads	Error (%)	Q20 (%)	Q30 (%)	GC (%)
**Female flowers – control**							
**2 DAT**	40,507,384	5,970,741,043	33,873,126 (83.62%)	0.0094	98.68	96.27	47.19
**4 DAT**	33,382,198	4,921,053,317	27,890,594 (83.55%)	0.0096	98.58	96.06	47.28
**10 DAT**	37,562,664	5,533,108,443	31,082,298 (82.75%)	0.0095	98.66	96.27	46.81
**Female flowers – GA_3 treatment**							
**2 DAT**	31,825,922	4,691,836,332	26,549,828 (83.42%)	0.0094	98.69	96.33	47.16
**4 DAT**	29,725,356	4,369,210,432	24,775,132 (83.35%)	0.0097	98.52	95.91	47.09
**10 DAT**	35,042,510	5,161,255,990	29,241,540 (83.45%)	0.0096	98.62	96.13	47.06
**Receptacle – control**							
**2 DAT**	32,072,882	4,725,438,484	26,724,548 (83.22%)	0.0094	98.68	96.3	47.74
**4 DAT**	33,984,708	4,999,991,286	28,394,554 (83.55%)	0.0096	98.61	96.11	47.7
**10 DAT**	39,066,466	5,751,772,897	32,078,648 (82.11%)	0.0095	98.63	96.15	47.38

**Receptacle – GA_3 treatment**							
**2 DAT**	36,232,880	5,330,791,349	30,257,864 (83.51%)	0.0096	98.61	96.12	47.71
**4 DAT**	32,486,194	4,778,371,956	27,252,842 (83.89%)	0.0096	98.59	96.06	47.84
**10 DAT**	31,798,676	4,681,066,843	26,272,570 (82.62%)	0.0096	98.62	96.16	47.32

DAT, days after treatment.

Table S3 Significant GO terms (corrected *P*-value ≤ 0.05) identified between control and GA_3-treated female flowers and receptacles

No.	GO terms	ID	Number of genes			
	**2 DAT**		**Female flowers**		**Receptacle**	
	**Molecular function**		**up**	**down**	**up**	**down**
1	electron carrier activity	GO:0009055	3	2	4	0
2	protein binding transcription factor activity	GO:0000988	0	1	2	0
3	binding	GO:0005488	139	94	156	135
4	transporter activity	GO:0005215	15	5	15	16
5	catalytic activity	GO:0003824	145	83	186	136
6	enzyme regulator activity	GO:0030234	3	7	4	4
7	molecular transducer activity	GO:0060089	4	1	5	3
8	receptor activity	GO:0004872	2	0	4	0
9	nucleic acid binding transcription factor activity	GO:0001071	23	3	15	12
10	antioxidant activity	GO:0016209	1	2	2	4
11	structural molecule activity	GO:0005198	0	0	1	4
	**Cellular component**					
1	symplast	GO:0055044	3	4	3	8
2	organelle	GO:0043226	33	45	40	65
3	cell part	GO:0044464	56	62	68	91
4	membrane part	GO:0044425	35	23	37	36
5	extracellular region	GO:0005576	3	4	9	8
6	cell junction	GO:0030054	3	4	3	8
7	membrane	GO:0016020	47	36	55	62
8	macromolecular complex	GO:0032991	30	27	34	36
9	cell	GO:0005623	56	62	68	91
10	organelle part	GO:0044422	8	29	14	29
	**Biological process**					
1	multiorganism process	GO:0051704	4	4	8	7
2	developmental process	GO:0032502	7	4	7	6
3	positive regulation of biological process	GO:0048518	4	1	1	1
4	multicellular organismal process	GO:0032501	7	4	10	5
5	cellular process	GO:0009987	141	83	158	135

6	single-organism process	GO:0044699	130	59	151	112
7	growth	GO:0040007	1	2	1	2
8	signaling	GO:0023052	11	3	11	7
9	negative regulation of biological process	GO:0048519	2	2	2	1
10	metabolic process	GO:0008152	182	117	215	192
11	biological regulation	GO:0065007	52	23	52	37
12	reproductive process	GO:0022414	2	2	4	3
13	regulation of biological process	GO:0050789	51	23	50	35
14	cellular component organization or biogenesis	GO:0071840	4	10	17	13
15	establishment of localization	GO:0051234	28	11	27	31
16	rhythmic process	GO:0048511	1	0	1	0
17	response to stimulus	GO:0050896	33	44	28	55
18	localization	GO:0051179	28	11	28	32
19	biological adhesion	GO:0022610	0	0	0	1
	**4 DAT**		**Female flowers**		**Receptacle**	
	**Molecular function**		**up**	**down**	**up**	**down**
1	electron carrier activity	GO:0009055	2	0	2	1
2	protein binding transcription factor activity	GO:0000988	0	0	2	1
3	binding	GO:0005488	37	73	159	121
4	transporter activity	GO:0005215	2	4	20	10
5	catalytic activity	GO:0003824	34	54	192	114
6	enzyme regulator activity	GO:0030234	1	1	3	3
7	molecular transducer activity	GO:0060089	0	2	3	2
8	structural molecule activity	GO:0005198	1	3	12	0
9	receptor activity	GO:0004872	0	0	2	0
10	nucleic acid binding transcription factor activity	GO:0001071	2	7	10	16
11	antioxidant activity	GO:0016209	0	2	1	0
	**Cellular component**					
1	symplast	GO:0055044	0	3	11	2
2	organelle	GO:0043226	8	24	69	38
3	cell part	GO:0044464	16	31	119	58
4	membrane part	GO:0044425	4	7	49	22
5	extracellular region	GO:0005576	8	1	15	2
6	cell junction	GO:0030054	0	3	11	2

7	membrane	GO:0016020	10	11	76	32
8	macromolecular complex	GO:0032991	3	17	56	28
9	cell	GO:0005623	16	31	119	58
10	organelle part	GO:0044422	4	9	35	17
	**Biological process**					
1	multiorganism process	GO:0051704	0	7	5	6
2	developmental process	GO:0032502	2	4	6	4
3	positive regulation of biological process	GO:0048518	1	2	3	2
4	multicellular organismal process	GO:0032501	2	6	10	4
5	cellular process	GO:0009987	30	64	190	100
6	single-organism process	GO:0044699	33	45	150	89
7	growth	GO:0040007	0	1	2	0
8	signaling	GO:0023052	0	4	15	0
9	negative regulation of biological process	GO:0048519	1	3	3	1
10	metabolic process	GO:0008152	39	84	227	150
11	biological regulation	GO:0065007	8	17	52	31
12	reproductive process	GO:0022414	1	6	4	5
13	regulation of biological process	GO:0050789	8	17	49	30
14	cellular component organization or biogenesis	GO:0071840	6	4	28	1
15	establishment of localization	GO:0051234	4	6	37	15
16	rhythmic process	GO:0048511	0	1	0	0
17	response to stimulus	GO:0050896	4	35	37	48
18	localization	GO:0051179	4	6	39	15
19	reproduction	GO:0000003	0	0	1	1
	**10 DAT**		**Female flowers**		**Receptacle**	
	**Molecular function**		**up**	**down**	**up**	**down**
1	electron carrier activity	GO:0009055	4	1	6	3
2	protein binding transcription factor activity	GO:0000988	1	0	1	1
3	binding	GO:0005488	191	278	198	250
4	transporter activity	GO:0005215	15	56	17	52
5	catalytic activity	GO:0003824	253	399	279	320
6	guanyl-nucleotide exchange factor activity	GO:0005085	1	0	1	0
7	enzyme regulator activity	GO:0030234	2	12	12	8
8	molecular transducer activity	GO:0060089	2	6	2	5

9	structural molecule activity	GO:0005198	14	0	9	2
10	receptor activity	GO:0004872	1	1	1	1
11	nucleic acid binding transcription factor activity	GO:0001071	15	20	1	1
12	antioxidant activity	GO:0016209	7	7	9	2
	**Cellular component**					
1	symplast	GO:0055044	7	4	11	6
2	extracellular matrix	GO:0031012	1	0	0	0
3	extracellular region part	GO:0044421	3	1	0	0
4	extracellular region	GO:0005576	0	0	24	11
5	organelle	GO:0043226	74	68	64	69
6	cell part	GO:0044464	108	109	106	109
7	membrane part	GO:0044425	33	88	48	74
8	extracellular region	GO:0005576	24	12	24	11
9	cell junction	GO:0030054	7	4	11	6
10	nucleoid	GO:0009295	0	1	0	0
11	membrane	GO:0016020	65	132	89	115
12	macromolecular complex	GO:0032991	49	48	38	49
13	cell	GO:0005623	108	109	106	109
14	organelle part	GO:0044422	39	32	27	32
	**Biological process**					
1	reproduction	GO:0000003	1	0	2	2
2	multiorganism process	GO:0051704	4	8	9	8
3	developmental process	GO:0032502	12	13	11	7
4	positive regulation of biological process	GO:0048518	4	4	1	5
5	multicellular organismal process	GO:0032501	12	10	12	8
6	cellular process	GO:0009987	188	290	203	263
7	single-organism process	GO:0044699	195	314	206	261
8	growth	GO:0040007	1	1	3	0
9	signaling	GO:0023052	9	15	9	16
10	negative regulation of biological process	GO:0048519	5	3	2	1
11	metabolic process	GO:0008152	280	432	300	363
12	biological regulation	GO:0065007	40	79	50	75
13	reproductive process	GO:0022414	3	2	5	5
14	regulation of biological process	GO:0050789	36	72	47	71
15	establishment of localization	GO:0051234	27	77	29	69

16	rhythmic process	GO:0048511	0	2	0	0
17	cellular component organization or biogenesis	GO:0071840	36	6	32	8
18	response to stimulus	GO:0050896	51	68	51	65
19	biological adhesion	GO:0022610	1	1	1	0
20	localization	GO:0051179	29	78	30	69

The representative terms with a large number of differentially expressed genes are highlighted in yellow. DAT, days after treatment.

Table S4 Significant KEGG pathways (corrected *P*-value ≤ 0.05) identified between GA_3-treated and control female flowers and receptacle

No.	Pathway	Number of genes	corrected *P*-value	Pathway ID
**Female flowers (2 DAT)**				
1	Protein processing in endoplasmic reticulum	34	2.87E-13	ko04141
2	Photosynthesis – antenna proteins	6	6.89E-04	ko00196
3	Photosynthesis	9	0.00217	ko00195
4	Plant–pathogen interaction	13	0.0224	ko04626
**Female flowers (4 DAT)**				
1	Protein processing in endoplasmic reticulum	25	1.02E-10	ko04141
**Female flowers (10 DAT)**				
1	Alanine, aspartate and glutamate metabolism	13	1.57E-06	ko00250
2	Phenylpropanoid biosynthesis	23	4.46E-04	ko00940
3	Phenylalanine metabolism	18	8.14E-04	ko00360
4	Flavonoid biosynthesis	10	0.00556	ko00941
5	Cysteine and methionine metabolism	15	0.00724	ko00270
6	Carbon fixation in photosynthetic organisms	14	0.0117	ko00710
7	Tryptophan metabolism	8	0.0143	ko00380
8	Arginine and proline metabolism	13	0.0143	ko00330
9	Galactose metabolism	11	0.0165	ko00052
10	Nitrogen metabolism	8	0.0170	ko00910
11	Fatty acid metabolism	9	0.0176	ko00071
12	Other glycan degradation	5	0.0191	ko00511
13	Tyrosine metabolism	8	0.0460	ko00350
**Receptacle (2 DAT)**				
1	Protein processing in endoplasmic reticulum	31	8.76E-08	ko04141
2	Photosynthesis – antenna proteins	7	3.90E-04	ko00196
3	Nitrogen metabolism	8	0.00257	ko00910
4	Phenylalanine metabolism	12	0.00888	ko00360
5	Plant hormone signal transduction	20	0.0150	ko04075
6	Cysteine and methionine metabolism	11	0.0150	ko00270
7	Glycine, serine and threonine metabolism	9	0.0154	ko00260
8	Carotenoid biosynthesis	6	0.0154	ko00906

9	Photosynthesis	8	0.0325	ko00195
**Receptacle (4 DAT)**				
1	Protein processing in endoplasmic reticulum	30	1.10E-06	ko04141
2	Circadian rhythm – plant	8	0.00231	ko04712
3	Cysteine and methionine metabolism	13	0.00281	ko00270
4	Endocytosis	13	0.0326	ko04144
**Receptacle (10 DAT)**				
1	Cysteine and methionine metabolism	21	2.95E-07	ko00270
2	Carbon fixation in photosynthetic organisms	15	9.82E-04	ko00710
3	Galactose metabolism	12	0.00229	ko00052
4	Phenylalanine metabolism	14	0.00863	ko00360
5	Pentose and glucuronate interconversions	12	0.0120	ko00040
6	Starch and sucrose metabolism	21	0.0120	ko00500
7	Plant hormone signal transduction	24	0.0120	ko04075
8	Tyrosine metabolism	8	0.0260	ko00350
9	Terpenoid backbone biosynthesis	8	0.0260	ko00900
10	Arginine and proline metabolism	11	0.0325	ko00330
11	Pentose phosphate pathway	8	0.0427	ko00030
12	Carotenoid biosynthesis	6	0.0460	ko00906

DAT, days after treatment.

Table S5 All gibberellin-, auxin- and cytokinin-synthesis, catabolism and response transcripts identified in this study which were differentially expressed (FDR < 0.01 and the absolute value of $\log_2$ ($FPKM_{treatment}/FPKM_{control}$) ≥ 1) in at least one pairwise comparison group

Gene name	Gene ID / Protein ID	Description in NR database	Similarity (%)
**Gibberellin metabolism- and response-related genes**			
**GA20ox**			
comp57463_c0	gi:587886862 / EXB75633.1	Gibberellin 20 oxidase 3 [*Morus notabilis*]	91%
**GA3ox**			
comp12680_c0	gi:587893059 / EXB81619.1	Gibberellin 3-beta-dioxygenase 4 [*Morus notabilis*]	88%
comp13433_c0	gi:587946228 / EXC32577.1	Gibberellin 3-beta-dioxygenase 1 [*Morus notabilis*]	89%
comp22862_c0	gi:587893059 / EXB81619.1	Gibberellin 3-beta-dioxygenase 4 [*Morus notabilis*]	89%
**GA2ox**			
comp16885_c0	gi:587973425 / EXC58248.1	Gibberellin 2-beta-dioxygenase 1 [*Morus notabilis*]	91%
comp26664_c0	gi:587946750 / EXC33074.1	Gibberellin 2-beta-dioxygenase 8 [*Morus notabilis*]	93%
comp32778_c0	gi:587942817 / EXC29353.1	Gibberellin 2-beta-dioxygenase [*Morus notabilis*]	93%
comp18866_c0	gi:587906001 / EXB94107.1	Gibberellin 2-beta-dioxygenase 8 [*Morus notabilis*]	80%
comp53019_c0	gi:587866532 / EXB55989.1	Gibberellin 2-beta-dioxygenase 2 [*Morus notabilis*]	85%
comp23223_c0	gi:587917544 / EXC05108.1	Gibberellin 2-beta-dioxygenase 8 [*Morus notabilis*]	93%
**DELLA**			
comp26619_c0	gi:645278651 / XP_008244327.1	PREDICTED: scarecrow-like protein 21 [*Prunus mume*]	80%

**Auxin metabolism- and response-related genes**			
**Aldehyde (IAld) dehydrogenase**			
comp9282_c0	gi:595802986 / XP_007201943.1	Hypothetical protein PRUPE_ppa004461mg [*Prunus persica*]	95%
comp11862_c0	gi:587899465 / EXB87864.1	Aldehyde dehydrogenase family 2 member [*Morus notabilis*]	82%
**IAA-amino acid hydrolase**			
comp20467_c0	gi:587844298 / EXB34859.1	IAA-amino acid hydrolase ILR1-like 4 [*Morus notabilis*]	96%
comp25015_c0	gi:587844301 / EXB34862.1	IAA-amino acid hydrolase ILR1-like 5 [*Morus notabilis*]	78%
comp12153_c0	gi:587894121 / EXB82653.1	IAA-amino acid hydrolase ILR1-like 2 [*Morus notabilis*]	95%
**GH3**			
comp25108_c0	gi:587861039 / EXB50903.1	Hypothetical protein L484_021129 [*Morus notabilis*]	96%
comp29694_c0	gi:587887925 / EXB76648.1	Hypothetical protein L484_011493 [*Morus notabilis*]	96%
**AUX1**			
comp30772_c0	gi:659131659 / XP_008465793.1	PREDICTED: auxin transporter-like protein 3 [*Cucumis melo*]	96%
**AUX/IAA**			
comp12280_c0	gi:587835799 / EXB26568.1	Auxin-induced protein IAA4 [*Morus notabilis*]	62%
comp16786_c0	gi:587939152 / EXC25824.1	Auxin-induced protein AUX22 [*Morus notabilis*]	82%
comp21990_c0	gi:587939153 / EXC25825.1	Auxin-responsive protein IAA17 [*Morus notabilis*]	87%
comp20539_c0	gi:388509620 / AFK42876.1	Unknown [*Medicago truncatula*]	77%
comp13087_c0	gi:587905040 / EXB93236.1	Auxin-responsive protein IAA3 [*Morus notabilis*]	84%
comp12423_c0	gi:587916406 / EXC04075.1	Auxin-responsive protein IAA13 [*Morus notabilis*]	86%

comp7006_c0	gi:470109762 / XP_004291159.1	PREDICTED: auxin-responsive protein IAA26-like [*Fragaria vesca* subsp. *vesca*]	68%
**ARF**			
comp32528_c0	gi:587869068 / EXB58397.1	Auxin response factor 5 [*Morus notabilis*]	95%
**SAUR**			
comp33019_c0	gi:587853002 / EXB43114.1	Hypothetical protein L484_002582 [*Morus notabilis*]	86%
comp36657_c0	gi:587929004 / EXC16179.1	Hypothetical protein L484_024347 [*Morus notabilis*]	95%
comp16559_c0	gi:587872564 / EXB61802.1	Hypothetical protein L484_012234 [*Morus notabilis*]	83%
comp31670_c0	gi:587846607 / EXB37077.1	Hypothetical protein L484_020868 [*Morus notabilis*]	92%
comp19373_c0	gi:587850151 / EXB40340.1	Hypothetical protein L484_017482 [*Morus notabilis*]	88%
**Cytokinin metabolism- and response-related genes**			
**IPT**			
comp9983_c0	gi:587907848 / EXB95832.1	Adenylate isopentenyltransferase 5 [*Morus notabilis*]	85%
**CKX**			
comp21589_c0	gi:587938282 / EXC25031.1	Cytokinin dehydrogenase 5 [*Morus notabilis*]	91%
comp11524_c0	gi:587923105 / EXC10466.1	Cytokinin dehydrogenase 1 [*Morus notabilis*]	95%
**CIS-ZOG**			
comp18972_c0	gi:587860262 / EXB50176.1	Zeatin O-glucosyltransferase [Morus notabilis]	88%
comp21293_c0	gi:587860262 / EXB50176.1	Zeatin O-glucosyltransferase [Morus notabilis]	80%

comp20461_c0	gi:604298708 / EYU18710.1	Hypothetical protein MIMGU_mgv1a005799mg [*Erythranthe guttata*]	86%
**CRE1**			
comp16591_c0	gi:587850113 / EXB40302.1	Histidine kinase 3 [*Morus notabilis*]	95%
**A-ARR**			
comp4260_c0	gi:587908595 / EXB96540.1	Two-component response regulator [*Morus notabilis*]	98%
comp16589_c0	gi:587945862 / EXC32234.1	Two-component response regulator [*Morus notabilis*]	88%

Table S6 All abscisic acid- and ethylene-synthesis, catabolism and response transcripts identified in this study which were differentially expressed (FDR < 0.01 and the absolute value of $\log_2$ ($FPKM_{treatment}/FPKM_{control}$) ≥ 1) in at least one pairwise comparison group

**Gene name**	**Gene ID / Protein ID**	**Description in NR database**	**Similarity (%)**
**ABA metabolism- and response-related genes**			
**NCED**			
comp22655_c0	gi:587942803 / EXC29339.1	Hypothetical protein L484_021647 [*Morus notabilis*]	82%
comp17110_c0	gi:587942803 / EXC29339.1	Hypothetical protein L484_021647 [*Morus notabilis*]	96%
comp26438_c0	gi:587930726 / EXC17835.1	Hypothetical protein L484_023189 [*Morus notabilis*]	89%
comp19377_c0	gi:587862256 / EXB52067.1	Hypothetical protein L484_024617 [*Morus notabilis*]	93%
**ABA2**			
comp12226_c0	gi:587923076 / EXC10437.1	Xanthoxin dehydrogenase [*Morus notabilis*]	93%
**ABA 8'-hydroxylase**			
comp9528_c0	gi:587945275 / EXC31689.1	Abscisic acid 8'-hydroxylase 1 [*Morus notabilis*]	94%
**PYR/PYL**			
comp25256_c0	gi:587862606 / EXB52402.1	Hypothetical protein L484_012047 [*Morus notabilis*]	92%
**PP2C**			
comp23225_c0	gi:587870483 / EXB59766.1	Putative protein phosphatase 2C 6 [*Morus notabilis*]	98%
comp25377_c0	gi:587848632 / EXB38891.1	Protein phosphatase 2C 16 [*Morus notabilis*]	90%
comp14332_c0	gi:587877015 / EXB66087.1	Putative protein phosphatase 2C 8 [*Morus notabilis*]	87%

comp16460_c0	gi:587923379 / EXC10729.1	Putative protein phosphatase 2C 24 [*Morus notabilis*]	85%
comp25508_c0	gi:645258105 / XP_008234728.1	PREDICTED: probable protein phosphatase 2C 51 [*Prunus mume*]	78%
comp19341_c0	gi:587933921 / EXC20871.1	Putative protein phosphatase 2C 75 [*Morus notabilis*]	78%
**ABF**			
comp30505_c0	gi:587946520 / EXC32855.1	ABSCISIC ACID-INSENSITIVE 5-like protein 5 [*Morus notabilis*]	91%
comp18261_c1	gi:375155309 / AFA37978.1	ABA responsive element-binding protein [*Solanum torvum*]	98%
comp16514_c0	gi:587946520 / EXC32855.1	ABSCISIC ACID-INSENSITIVE 5-like protein 5 [*Morus notabilis*]	87%
comp89455_c0	gi:587848159 / EXB38447.1	ABSCISIC ACID-INSENSITIVE 5-like protein 1 [*Morus notabilis*]	86%
**Ethylene metabolism- and response-related genes**			
**SAM synthetase**			
comp11492_c0	gi:255556894 / XP_002519480.1	S-adenosylmethionine synthetase, putative [*Ricinus communis*]	100%
comp28987_c0	gi:460408090 / XP_004249481.1	PREDICTED: S-adenosylmethionine synthase 2-like [*Solanum lycopersicum*]	100%
comp28816_c0	gi:449451048 / XP_004143274.1	PREDICTED: S-adenosylmethionine synthase 2-like [*Cucumis sativus*]	100%
comp30786_c0	gi:255543014 / XP_002512570.1	S-adenosylmethionine synthetase, putative [*Ricinus communis*]	100%
comp7483_c0	gi:470132951 / XP_004302338.1	PREDICTED: S-adenosylmethionine synthase 1-like [*Fragaria vesca* subsp. *Vesca*]	98%
**ACC synthase**			
comp27593_c0	gi:587846852 / EXB37292.1	1-Aminocyclopropane-1-carboxylate synthase [*Morus notabilis*]	94%
comp21829_c0	gi:587861793 / EXB51626.1	1-Aminocyclopropane-1-carboxylate synthase 1 [*Morus notabilis*]	95%
**ACC oxidase**			

comp23635_c1	gi:166788505 / BAG06705.1	1-Aminocyclopropane-1-carboxylate oxidase [*Ficus carica*]	100%
comp27433_c0	gi:587864354 / EXB54016.1	1-Aminocyclopropane-1-carboxylate oxidase-3-like protein [*Morus notabilis*]	90%
comp30808_c0	gi:587929255 / EXC16422.1	1-Aminocyclopropane-1-carboxylate oxidase-1-like protein [*Morus notabilis*]	86%
comp9988_c0	gi:587870860 / EXB60136.1	1-Aminocyclopropane-1-carboxylate oxidase 5 [*Morus notabilis*]	94%
comp11309_c0	gi:587940644 / EXC27244.1	1-Aminocyclopropane-1-carboxylate oxidase 3 [*Morus notabilis*]	97%
comp585_c0	gi:645235671 / XP_008224379.1	PREDICTED: 1-Aminocyclopropane-1-carboxylate oxidase homolog 1-like [*Prunus mume*]	91%
**ETR**			
comp16652_c0	gi:587910281 / EXB98165.1	Ethylene receptor 2 [*Morus notabilis*]	92%
comp29504_c0	gi:587907745 / EXB95735.1	Ethylene response sensor 1 [*Morus notabilis*]	96%
comp30741_c0	gi:587905004 / EXB93200.1	Protein EIN4 [*Morus notabilis*]	94%
**EBF1/2**			
comp9018_c0	gi:587830385 / EXB21298.1	Hypothetical protein L484_002248 [*Morus notabilis*]	95%
**ERF**			
comp16781_c0	gi:587914274 / EXC02053.1	Ethylene-responsive transcription factor 2 [*Morus notabilis*]	82%
comp16998_c0	gi:587864373 / EXB54035.1	Ethylene-responsive transcription factor 1B [*Morus notabilis*]	94%
comp17108_c0	gi:587854580 / EXB44623.1	Ethylene-responsive transcription factor 4 [*Morus notabilis*]	82%
comp28870_c0	gi:225455041 / XP_002282167.1	PREDICTED: Ethylene-responsive transcription factor 5 [*Vitis vinifera*]	60%

comp10854_c0	gi:587944083 / EXC30582.1	Ethylene-responsive transcription factor 12 [*Morus notabilis*]	84%
comp22612_c0	gi:587864369 / EXB54031.1	Ethylene-responsive transcription factor [*Morus notabilis*]	83%
comp25440_c0	gi:587847245 / EXB37641.1	Ethylene-responsive transcription factor 13 [*Morus notabilis*]	79%
comp46255_c0	gi:587945546 / EXC31946.1	Ethylene-responsive transcription factor 15 [*Morus notabilis*]	76%
comp7982_c0	gi:587892625 / EXB81200.1	Ethylene-responsive transcription factor [*Morus notabilis*]	90%
comp24422_c0	gi:587925050 / EXC12328.1	Ethylene-responsive transcription factor [*Morus notabilis*]	64%
comp18924_c0	gi:587925050 / EXC12328.1	Ethylene-responsive transcription factor [*Morus notabilis*]	74%
comp33022_c0	gi:587925344 / EXC12612.1	AP2-like ethylene-responsive transcription factor ANT [*Morus*	90%
comp7744_c0	gi:587934118 / EXC21056.1	Ethylene-responsive transcription factor 2 [*Morus notabilis*]	81%
comp34364_c0	gi:587935540 / EXC22410.1	AP2-like ethylene-responsive transcription factor ANT [*Morus*	85%
comp39079_c0	gi:587892747 / EXB81318.1	Ethylene-responsive transcription factor 3 [*Morus notabilis*]	86%

Supplementary Figure legends

Figure S1 Verification of RNA-Seq results by qRT-PCR. Bars represent standard deviation. F, female flowers; R, receptacle

Figure S2 Correlation of fold changes in gene expression between RNA-Seq and qRT-PCR. Equation of linear regression and correlation coefficient (R^2) are shown. DAT, days after treatment

Figure S3 Number of differentially expressed genes (FDR ≤ 0.001 and $\log_2(FPKM_{treatment}/FPKM_{control}) \geq 1$ or ≤ -1) between control and GA_3-treated female flowers and receptacles. DAT, days after treatment

Figure S4 Heat maps of hormone-related genes with low expression. FPKM of all samples was < 10

Figure S1

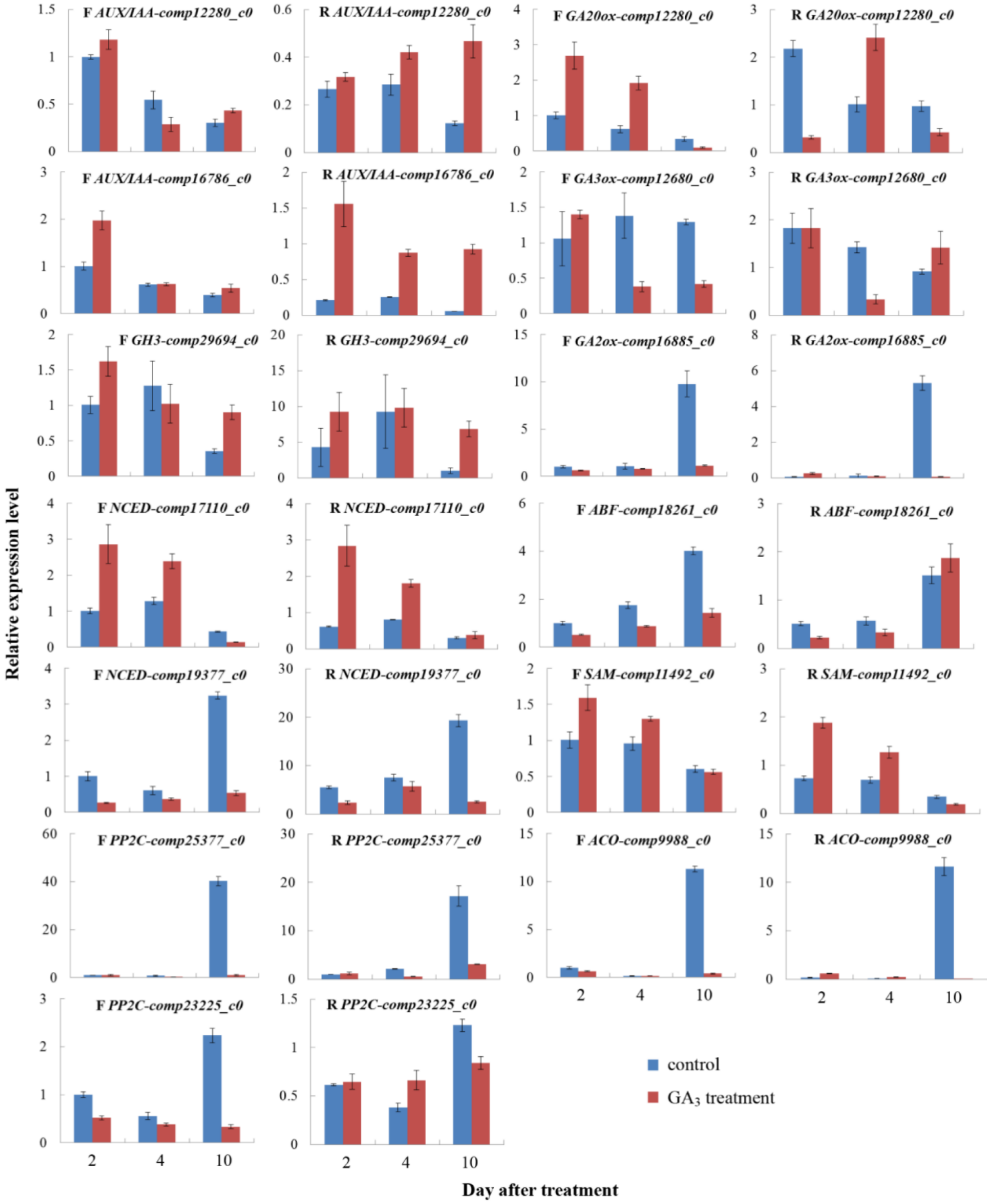
F AUX/IAA-comp12280_c0
R AUX/IAA-comp12280_c0
F GA20ox-comp12280_c0
R GA20ox-comp12280_c0
F AUX/IAA-comp16786_c0
R AUX/IAA-comp16786_c0
F GA3ox-comp12680_c0
R GA3ox-comp12680_c0
F GH3-comp29694_c0
R GH3-comp29694_c0
F GA2ox-comp16885_c0
R GA2ox-comp16885_c0
F NCED-comp17110_c0
R NCED-comp17110_c0
F ABF-comp18261_c0
R ABF-comp18261_c0
F NCED-comp19377_c0
R NCED-comp19377_c0
F SAM-comp11492_c0
R SAM-comp11492_c0
F PP2C-comp25377_c0
R PP2C-comp25377_c0
F ACO-comp9988_c0
R ACO-comp9988_c0
F PP2C-comp23225_c0
R PP2C-comp23225_c0
Relative expression level
Day after treatment
control
GA3 treatment

Figure S2

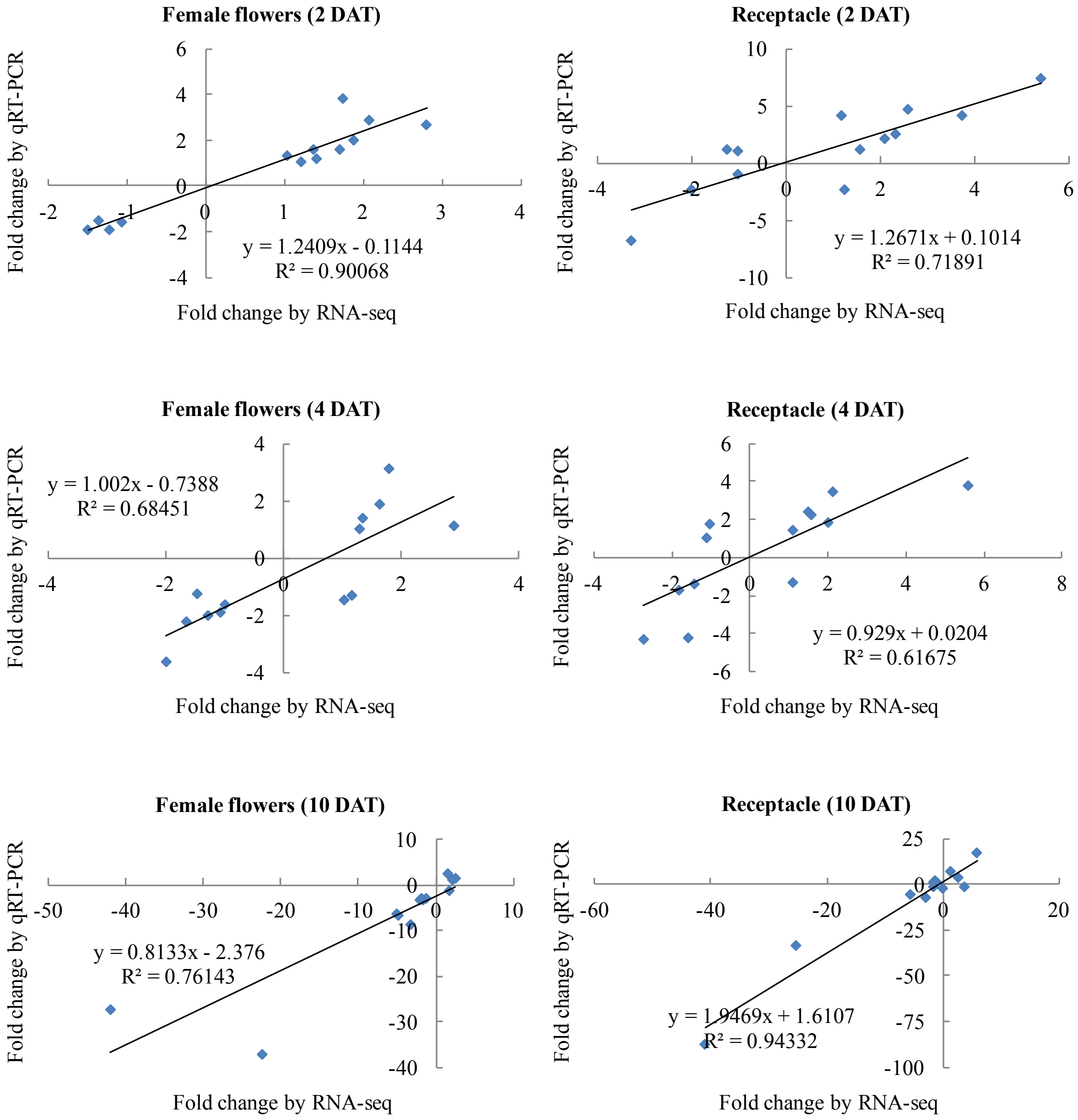

Female flowers (2 DAT)
Fold change by qRT-PCR
Fold change by RNA-seq
y = 1.2409x - 0.1144
R² = 0.90068
Receptacle (2 DAT)
Fold change by qRT-PCR
Fold change by RNA-seq
y = 1.2671x + 0.1014
R² = 0.71891
Female flowers (4 DAT)
Fold change by qRT-PCR
Fold change by RNA-seq
y = 1.002x - 0.7388
R² = 0.68451
Receptacle (4 DAT)
Fold change by qRT-PCR
Fold change by RNA-seq
y = 0.929x + 0.0204
R² = 0.61675
Female flowers (10 DAT)
Fold change by qRT-PCR
Fold change by RNA-seq
y = 0.8133x - 2.376
R² = 0.76143
Receptacle (10 DAT)
Fold change by qRT-PCR
Fold change by RNA-seq
y = 1.9469x + 1.6107
R² = 0.94332

Figure S3

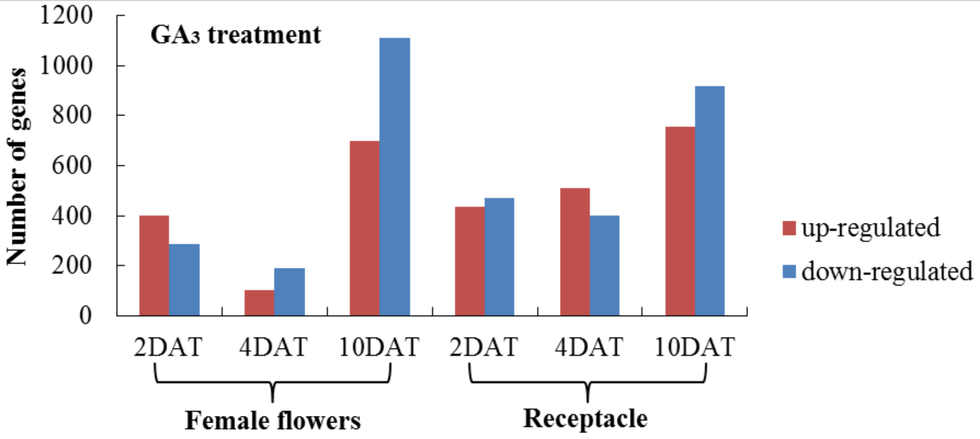

GA3 treatment
Number of genes
1200
1000
800
600
400
200
0
2DAT
4DAT
10DAT
2DAT
4DAT
10DAT
Female flowers
Receptacle
up-regulated
down-regulated

Figure S4

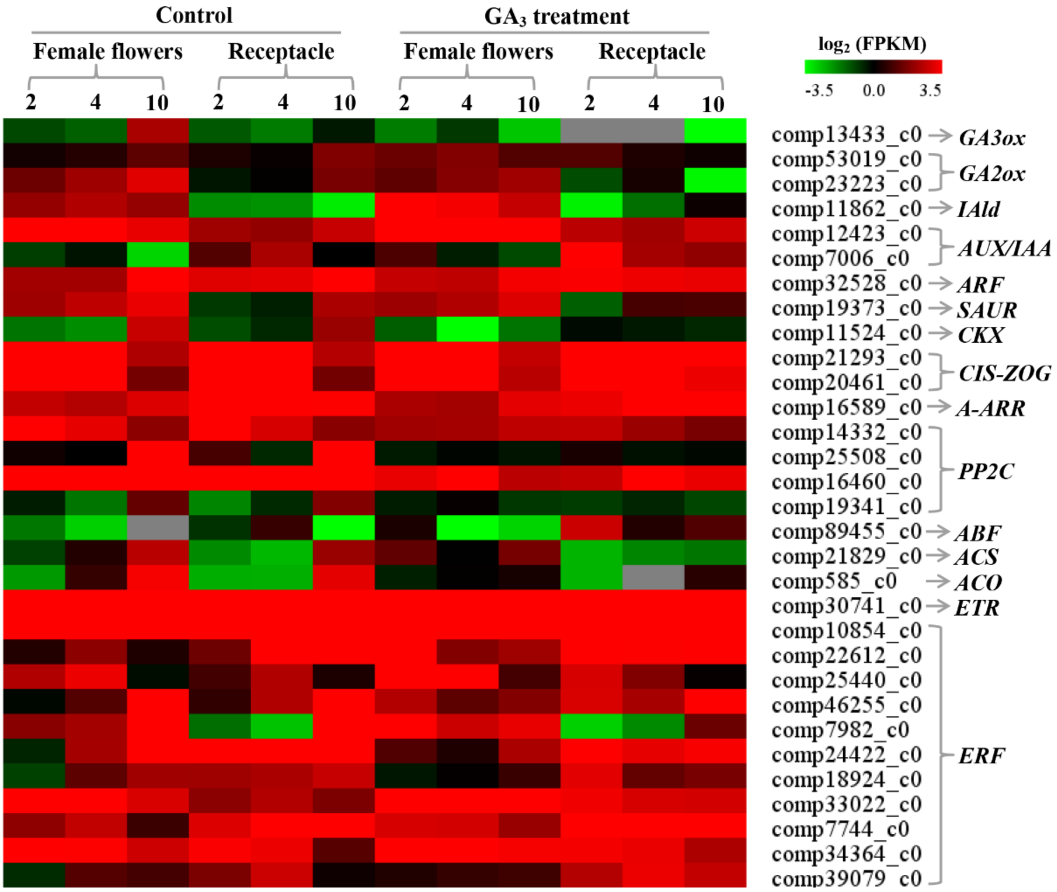

Control
GA3 treatment
Female flowers
Receptacle
Female flowers
Receptacle
2
4
10
2
4
10
2
4
10
2
4
10
log2 (FPKM)
-3.5
0.0
3.5
comp13433_c0 → GA3ox
comp53019_c0
comp23223_c0
GA2ox
comp11862_c0 → IAld
comp12423_c0
comp7006_c0
AUX/IAA
comp32528_c0 → ARF
comp19373_c0 → SAUR
comp11524_c0 → CKX
comp21293_c0
comp20461_c0
CIS-ZOG
comp16589_c0 → A-ARR
comp14332_c0
comp25508_c0
comp16460_c0
comp19341_c0
PP2C
comp89455_c0 → ABF
comp21829_c0 → ACS
comp585_c0 → ACO
comp30741_c0 → ETR
comp10854_c0
comp22612_c0
comp25440_c0
comp46255_c0
comp7982_c0
comp24422_c0
comp18924_c0
comp33022_c0
comp7744_c0
comp34364_c0
comp39079_c0
ERF
